# Comparative Ungulate Diversity and Biomass Change With Human Use and Drought: Implications for Community Stability and Protected Area Prioritization in African Savannas

**DOI:** 10.1002/ece3.71946

**Published:** 2025-08-28

**Authors:** Gundula S. Bartzke, Joseph O. Ogutu, Hans‐Peter Piepho, Claire Bedelian, Michael E. Rainy, Russel L. Kruska, Jeffrey S. Worden, Kamau Kimani, Michael J. McCartney, Leah Ng'ang'a, Jeniffer Kinoti, Evanson C. Njuguna, Cathleen J. Wilson, Richard Lamprey, Nicholas Thompson Hobbs, Robin S. Reid

**Affiliations:** ^1^ Biostatistics Unit, Faculty of Agricultural Sciences, Institute of Crop Science University of Hohenheim Stuttgart Baden‐Württemberg Germany; ^2^ International Livestock Research Institute Nairobi Nairobi County Kenya; ^3^ Danish Institute for International Studies Copenhagen Capital Region of Denmark Denmark; ^4^ World Wildlife Fund Nairobi Nairobi County Kenya; ^5^ Campfire Conservation Nairobi Nairobi County Kenya; ^6^ Department of Infrastructure, Lands and Urban Development County Government of Laikipia Rumuruti Laikipia Kenya; ^7^ Department of Natural Resources, Faculty of Geo‐Information Science and Earth Observation University of Twente Enschede Overste the Netherlands; ^8^ Natural Resource Ecology Laboratory, Department of Ecosystem Science and Sustainability Colorado State University Fort Collins Colorado USA

**Keywords:** biomass, disturbance, diversity, drought, savanna, ungulates

## Abstract

Drought and human use may alter ungulate diversity and biomass in contrasting ways. In African savannas, resource‐dependent grazers such as wildebeest ( *Connochaetes taurinus* ) and zebra ( *Equus quagga* ) may decline or disperse as resources decline, opening space for more drought‐tolerant species such as gazelles (*Eudorcas* and *Nanger*) and impala ( *Aepyceros melampus* ). This shift can increase species richness, evenness, and overall ungulate diversity. Although higher diversity may stabilize ungulate communities, it may be associated with lower biomass (the total body mass of all individuals in a community), which in turn affects vegetation structure and composition, nutrient cycling, energy flows, and other organisms in savannas. While ungulate biomass often declines during drought or in areas of intense human use, the effects on diversity changes under low‐to‐moderate human use remain less clear. Our fine‐scale censuses in the Maasai Mara National Reserve and adjacent pastoral lands in Kenya showed that ungulate biomass declined more than diversity in the 1999 drought year. In the normal rainfall year of 2002, diversity peaked along the reserve boundary, but species richness leveled off in the drought year. Biomass peaked in the reserve in both census years, and migratory ungulates moved further into the reserve in the drought year, where diversity declined. These findings suggest that core protected areas are crucial for maintaining ungulate *biomass*, while transition zones from protected and pastoral lands support higher *diversity* unless drought reduces species richness.

## Graphical abstract

This study describes how the diversity and biomass of African savanna ungulates change with drought and human use in the Maasai Mara Ecosystem in Kenya. The results suggest that core protected areas are crucial for maintaining ungulate biomass. Meanwhile, transition zones between protected areas and pastoral lands can support higher ungulate diversity, unless drought reduces species richness.

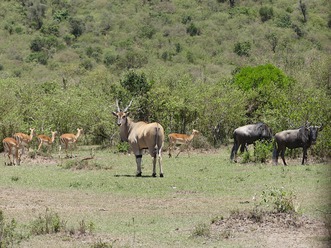

## 1 Introduction

Diversity and biomass are key components of biological communities. Declines in the abundance of individual species reduce biomass but do not necessarily diminish diversity unless those species go extinct or rare species decline disproportionately. Indeed, biomass can decrease while species diversity increases (Grime and Pierce 2012) if common species suffer more substantial declines, thereby freeing resources for rare species and creating a richer or more even community (Connell 1978). Focusing conservation efforts solely on the least common species may fail to maintain overall biomass dominated by common species. Conversely, concentrating conservation efforts on common species may neglect potential threats to rare species and diversity. Balancing the needs of different species is challenging due to varying ecological requirements. Addressing this complexity requires attention not only to individual species' needs but also to the processes that shape biomass and diversity.

Diversity, with its intrinsic value and underlying contribution to community stability (Tilman and Downing 1994; Olivier et al. 2020), may be especially important in African savannas. Ungulate functional diversity, encompassing grazers, browsers, and mixed feeders, controls vegetation growth and structure (Dublin et al. 1990; Sinclair 1995; McNaughton 1984) and supports a diverse carnivore community through a range of prey sizes (Sinclair et al. 2003). Certain ungulate species also aid in seed dispersal (Miller 1996; Bunney et al. 2017). Both the abundance and diversity of large mammals seem to attract millions of tourists to the African savanna (Lindsey et al. 2007; Arbieu et al. 2018), generating local and national economic benefits (Thirgood et al. 2008; World Tourism Organization 2019). Consequently, changes in ungulate diversity should concern conservationists for both economic and ecological reasons.

A sufficient level of ungulate biomass is important for maintaining savanna vegetation and preventing transitions to shrubland or woodland (Dublin et al. 1990; Sinclair 1995). High ungulate biomass also accelerates nutrient cycling through dung, urine, or carrion (McNaughton et al. 1988), influences mineralization of plant‐available nitrogen by soil microbes (Seagle et al. 1992), and promotes carbon‐based energy flow from plants to animals in savannas (Detling 1988). While ungulates benefit some small animal species (Ogada et al. 2008; Nasseri et al. 2011), they can negatively affect others (Pringle et al. 2007; Long et al. 2017; Guy et al. 2021) by trampling vegetation (Cumming and Cumming 2003) and reducing trees, herbs (Pringle et al. 2007), and flowers. Notably, bird species richness peaked at intermediate rather than low or high ungulate presence (Francis et al. 2020), suggesting that savanna conservation should balance the benefits and drawbacks of high ungulate biomass.

**FIGURE 1:**
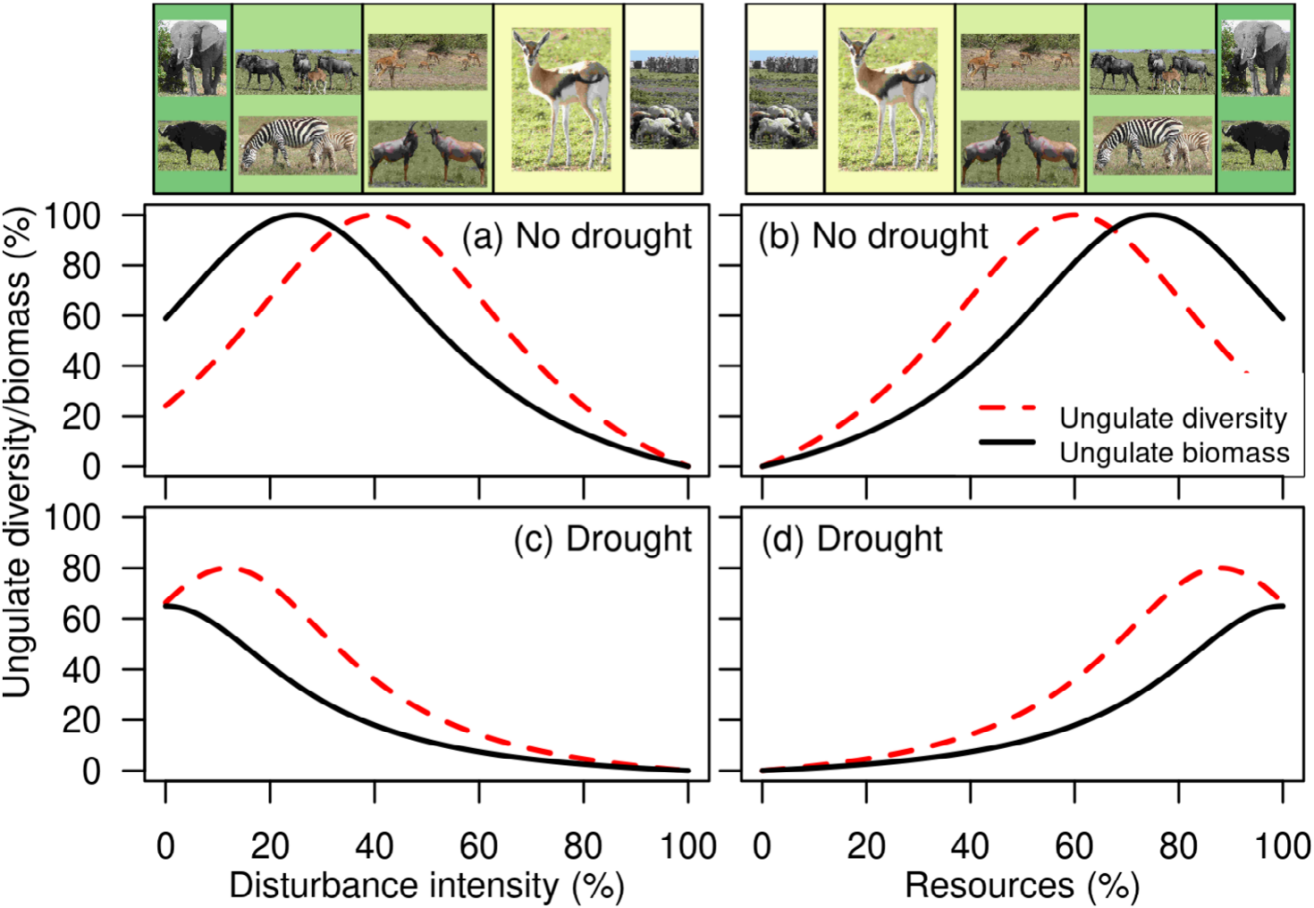
Hypothetical change in peak diversity (red dotted lines) and peak biomass (black lines) of African savanna ungulates during normal rainfall (a, b) and drought (c, d) as a function of disturbance intensity (a, c) and resources (b, d). Photos by Gundula S. Bartzke.

Although ungulate abundance and diversity can decline with increasing human use near protected area boundaries (Green et al. 2019), it is uncertain how these community attributes respond under low‐to‐moderate human use, such as livestock grazing (Xu and Butt 2024; Ogutu et al. 2025). Typically, ungulate biomass increases with rainfall (Coe et al. 1976), whereas ungulate diversity peaks at intermediate rainfall (Faith 2013). This pattern suggests that as human use affects vegetation and water resources, these community attributes may change in different ways. Livestock grazing, for example, removes tall grass preferred by large herbivores such as the African elephant ( *Loxodonta africana* ) but can promote young grass growth (Charles et al. 2017), which benefits often smaller herbivores such as impala ( *Aepyceros melampus* ; Figure 1a).

Shrub encroachment, livestock grazing, or fire tied to human uses (Roques et al. 2001) are often associated with declines in mammalian functional diversity (Chillo and Ojeda 2012). By contrast, the Intermediate Disturbance Hypothesis predicts that diversity should peak at intermediate disturbance frequencies or intensities (Connell 1978; Figure 1a) because such disturbances can open up sites for subordinate species by reducing the abundance of dominant species (Connell 1978). Similarly, the humped‐back model of species richness and biomass production predicts that the highest species richness occurs at intermediate productivity environments, where more spatial and temporal niches are available (Grime and Pierce 2012, Figure 1a). These concepts have broad applications in explaining the diversity of plants and some small animal communities (Pierce 2014; Connell 1978; Battisti and Fanelli 2016; Winfree et al. 2007), yet they are less established for large animal communities (Mackey and Currie 2001; Appendix A; Owen 1990).

Although ungulate abundance, species richness, and evenness have been linked to several resource and human use variables (Green et al. 2019; Crego et al. 2020; Kinnaird and O'brien 2012), the influence of drought on these relationships is rarely quantified (Ogutu et al. 2010). Drought severity, frequency, and time since drought can define gradients in disturbance intensity (Figure 1a). However, without repeated measurements, drought may mask or alter diversity changes along continuous gradients of disturbance or resource availability (Figure 1a–d). After drought, the disturbance optimum could shift to lower disturbance and higher resource levels compared with normal rainfall conditions, mirroring systems of high or low productivity (Figure 1c,d; Seidl et al. 2022).

We hypothesized that a severe drought, such as the one that occurred in the Maasai Mara ecosystem in 1999, would cause an immediate overall decline in ungulate diversity and biomass. We also expected that peaks in ungulate diversity and biomass would shift toward lower intense human use and more available resources when they become scarce (Figure 1a,b), as animals concentrate in resource‐rich areas during scarcity (Anderson et al. 2012). Furthermore, we predicted that ungulate biomass would be more sensitive to human disturbances or resource reductions (Figure 1a,b), given that roughage feeders, such as African elephant ( *Loxodonta africana* ), buffalo ( *Syncerus caffer* ), zebra ( *Equus quagga* ), and wildebeest ( *Connochaetes taurinus* , Cumming 1982) are particularly vulnerable to drought‐induced resource shortages (Abraham et al. 2019; Okello et al. 2016; Dublin and Ogutu 2015). They can also move more easily to higher resource areas than small herbivores or browsers (Abraham et al. 2019).

Browsers such as the giraffe ( *Giraffa camelopardalis* ) or mixed feeders such as gazelles (*Eudorcas* and *Nanger*) or impala ( *Aepyceros melampus* ) can cope better with deficits in resources such as grass (Abraham et al. 2019) and, except for the giraffe, limited water availability (Kihwele et al. 2020). Yet they contribute less to biomass (Cumming 1982). Although complete disturbance or absolute resource scarcity may be rare in pastoral landscapes, we include them in the full spectrum of possible gradients ( *Aepyceros melampus* ; Figure 1a,b). In areas of intense human use, livestock may be the only ungulates remaining after wild species have departed (Figure 1a,b).

To verify these predictions, we have compared ungulate diversity and biomass in a drought year (1999) and a normal rainfall year (2002) under protection in the Maasai Mara National Reserve (“Mara Reserve”) and under pastoralism in adjacent areas in the Greater Mara Ecosystem in Kenya. We censused and mapped the entire large mammal assemblage at a fine scale. Our analysis builds on previous studies that have focused on changes in the densities of common wild and domestic ungulate species relative to distance from water points and settlements and that have compared the densities of individual ungulate species in the reserve and on pastoral lands (Ogutu et al. 2010, 2014; Bhola et al. 2012). Here, we specifically investigate how ungulate diversity and biomass vary with distance from the reserve boundary in this savanna landscape. We also identify other potentially important predictors of ungulate diversity and biomass from a set of 26, sometimes intercorrelated, variables.

## 2 Methods

### 2.1 Data Collection

#### 2.1.1 Study Area

**FIGURE 2:**
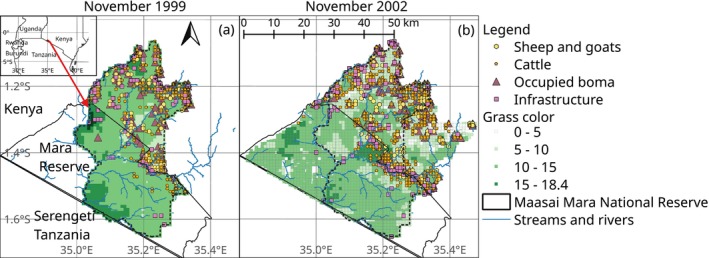
Locations of sheep and goats (yellow diamonds), cattle (orange circles), occupied bomas (dark purple triangles), and other infrastructure (purple squares) in the Maasai Mara National Reserve (black polygon) and adjacent pastoral lands in Kenya in November of the drought year 1999 and November of the normal rainfall year 2002.

Our study area included about half the Maasai Mara National Reserve and similarly sized adjacent pastoral lands (85%; Figure 2). We focused on the overlapping study area censused in 1999 and 2002, covering 1475 km^2^ (Figure 2; Appendix S1: Figure S1). Droughts similar to the one in 1999 occur less than once every 10 years in the reserve (Bartzke et al. 2018). In 2002, total rainfall was close to the long‐term average of 986 mm (Bartzke et al. 2018). The reserve borders the Serengeti National Park to the south (Figure 2). The Serengeti‐Mara ecosystem supports about 1.3 million migrating wildebeest, 440,000 Thomson's gazelle ( *Eudorcas thomsonii nasalis* ) and 200,000 zebra, numerous other ungulates and carnivores (Sinclair et al. 2008). Seasonal and spatial rainfall gradients control vegetation and water availability for ungulates (Norton‐Griffiths et al. 1975; Boutton et al. 1988; Dessu et al. 2014; Ogutu et al. 2008). The cumulative rainfall during the 1999 wet season (range: 499–764 mm) was 16%–26% lower than that during the 2002 wet season (range: 677–906 mm). In contrast, dry season rainfall in 1999 (range: 147–337 mm) was 4%–29% higher than that in 2002 (range: 142–262 mm).

In the Mara Reserve, vegetation consists of grasses, scattered shrubs, and trees that transition to grazed pastoral land with similar or higher shrub and tree cover (Appendix S1: Figures S2 and S3m,p,s). Although livestock grazing and pastoral settlements, bomas, are not allowed in the reserve, we observed livestock up to 7.1 km into the reserve during data collection (Figure 2a,b). Bomas are huts with thatched grass, dung, or tin roofs (Reid et al. 2003). These enclosures have thorns to protect the livestock from predators (Ogutu et al. 2014). Bomas almost always occurred on pastoral land (Figure 2a,b). The level of human use in the area varies from low in the interior of the reserve to intermediate along the boundary, to high in the more distant pastoral areas (Figure 2a,b). Grass color, an indicator of resources for ungulates, fades from within the reserve to the pastoral lands (Figure 2a,b). Higher rainfall in areas far from the reserve may have reversed this trend in 1999 (Appendix S1: Figure S5).

Since 2005, pastoral communities, NGOs, government, and tourism operators have established community wildlife conservancies on formerly communal group ranches adjacent to the Maasai Mara National Reserve (Maasai Mara Wildlife Conservancies Association (MMWCA) 2019). This initiative aimed to mitigate land use changes such as settlement, fencing, and agriculture that have intensified in the Kenyan Mara (Veldhuis et al. 2019; Løvschal et al. 2022). Our data were collected in two sampling sessions in 1999 and 2002, before these conservancies were established, and the number of settlements roughly doubled in the 2000s (Veldhuis et al. 2019). Although the situation is different today, these valuable data can clarify the ecological processes shaping the diversity and biomass of African savanna ungulates.

#### 2.1.2 Field Data Collection

In 1999 and 2002, data on ungulate diversity and biomass were collected by teams coordinated by the International Livestock Research Institute (Reid et al. 2003; Ogutu et al. 2010, 2014). They censused 26,807 sub‐blocks measuring 333 m‐by‐333 m nested within 1667 blocks of 1 km‐by‐1 km. Each 1 km‐by‐1 km block contained nine such sub‐blocks or sampling units (Reid et al. 2003). The fieldwork, conducted by 12 teams of 40 people in 1999 and 22 teams of 84 people in 2002 over 5 days, could have taken a single person 620 days. The scale and cost of the fieldwork allowed only one sample from a drought year and one from a normal rainfall year. Although multiple years of sampling would be necessary to fully understand long‐term changes in ungulate diversity and biomass in relation to rainfall variability, this study produced the most detailed spatial data to date from the Mara‐Serengeti ecosystem under contrasting environmental conditions. The sampling focused on resident ungulates after most of the migrating wildebeest (Pennycuick 1975; Thirgood et al. 2004; Boone et al. 2006), and zebra had returned to the Serengeti. Although wildebeest numbers can be high in the northern Serengeti‐Mara Ecosystem during October and November in some years (Pennycuick 1975), telemetry data from 1999 to 2002 indicate that they had largely left the reserve by the time we collected data in November (Thirgood et al. 2004; Boone et al. 2006).

Team members counted wild ungulates, livestock, and carnivores, and recorded vegetation characteristics and other landscape features such as bomas, other buildings, fences, fields, old and recent fire scars, water sources, vehicles, and scattered litter in each sub‐block (Reid et al. 2003; Ogutu et al. 2010, 2014). Abandoned bomas, identified up to about 20 years after abandonment by their bright circular greenness caused by nutrient inputs from accumulated livestock dung (Muchiru et al. 2009), were also documented. Vegetation percent cover, height, and color were estimated visually: grasses were sampled using 2 m‐by‐2 m plots within three sub‐plots of each block, while shrubs and trees were assessed only in the central sub‐block (Reid et al. 2003). Inaccessible areas due to dense shrubs, deep swamps, or steep hills were mapped but not sampled. Soil type was not included as a predictor, though variation in soil nutrient‐fixing capacity may influence ungulate diversity (East 1984) and biomass; its effect was captured by vegetation variables recorded in the field. Variation in vegetation greenness and cover related to soil types was recorded during fieldwork. Environmental variables such as distance to water, dry season rainfall, grass height, shrub cover, wet season rainfall, preceding month's rainfall, and grass color differed between the drought and normal rainfall years (Appendix S1: Figures S2 and S3d,h–j,n–p).

### 2.2 Data Preparation

We prepared the data separately for each of the 2 years (https://doi.org/10.5281/zenodo.16884346: file “Metadata.pdf,” data file “mc_333m.csv,” R code “prepare_data.r”) but for brevity we explain the procedures for only 1 year.

#### 2.2.1 Imputing Missing Vegetation Observations

To predict grass, shrub, and tree cover, height, and color for unsampled sub‐blocks due to the sampling design, we applied automated kriging as described in Appendix S2: Section S1. This procedure used vegetation data from sampled sub‐blocks to estimate variances and spatial covariances based on variograms; then produced estimates for the unsampled sub‐blocks (Appendix S2: Section S1).

#### 2.2.2 Estimating Ungulate Abundances

At the 333 m‐by‐333 m sub‐block scale, 73% of observations in 1999 and 63% in 2002 were zeros. To reduce this high proportion of zeros to 23% in 1999 and 15% in 2002, we derived ungulate abundances at the 1 km‐by‐1 km block level (Appendix S1: Figure S1a–d). Blocks sometimes contained fewer than nine sub‐blocks because of edges or inaccessibility (18% in 1999 and 29% in 2002). To avoid underestimating block totals, we approximated the expected total number of individuals per block and species combination by

(1) $$c_{\mathrm{ui}}=\frac{\sum_{j}^{{\left|Q\right|}_{i}}d_{\mathrm{uij}}\times 9}{{\left|Q\right|}_{i}}$$

where ${\left|Q\right|}_{i}$ is the number of sub‐blocks in the *i*'th block and *d_uij_* is the number of individuals of the *u*'th ungulate species in the *j*'th sub‐block. This allowed us to estimate the expected ungulate numbers for blocks with missing sub‐blocks.

#### 2.2.3 Estimating Ungulate Diversity

Some ungulate species might have been missed in tall grass or high shrub cover, potentially biasing the raw species richness *S*i (including zero observations). To account for this, we used the R package entropart version 1.6–13 (Marcon and Hérault 2015) in R statistical software version 4.4.0 (R Core Team 2022) to estimate bias‐adjusted species richness ^0^ *D*_*i*_ for all blocks with observed ungulates. This jackknife estimator determined observation frequencies of each species using sequential statistical tests (Burnham and Overton 1979; Appendix S2: Section S2). Species richness corresponds to diversity ^*q*^ *D*_*i*_ of order *q* = 0 (Hill 1973; Marcon and Hérault 2015).

To quantify ungulate diversity beyond richness, we derived the bias‐adjusted effective number of species based on diversity orders 1 (transformed Shannon entropy; Shannon 1948), 2 (reciprocal Simpson index; Simpson 1949) and 10 (species evenness; Appendix S2: Section S2). Higher diversity orders weigh evenness more heavily than richness. The upper bound, ^∞^ *D*_*i*_, represents only evenness in terms of the reciprocal Berger–Parker index (Hill 1973; Berger and Parker 1970), but could not be estimated for blocks in which multiple species tied for top abundance. Estimates of the effective number of ungulate species depended on rank abundance and the total number of species in each block (Appendix S2: Section S2).

#### 2.2.4 Estimating Ungulate Biomass

We estimated ungulate biomass by multiplying the total number of each ungulate species in each block by its unit weight (Coe et al. 1976) and summing individuals of these products. To analyze potential effects of migration on biomass trends, we also calculated the biomass of migratory (wildebeest and zebra) and nonmigratory species. Estimates of ungulate biomass may have been affected by reduced visibility, albeit to a lesser extent than estimates of raw species richness. Small groups and species are more easily overlooked (Jachmann 2002; Rouhbakhsh et al. 2025), but they should contribute less to biomass than larger ones.

#### 2.2.5 Extracting Seasonal and Monthly Rainfall

To relate ungulate diversity and biomass to rainfall variability, we extracted monthly rainfall from the CHIRPS dataset (Funk et al. 2015) at each sub‐block's center. Different components of rainfall can influence ungulate communities in various ways (Appendix A: Rainfall Components). We considered rainfall during the wet and dry seasons, as well as in the preceding month. The wet season spans from November of the previous year to May of the survey year, and the dry season spans from June to October of the survey year.

#### 2.2.6 Landscape Variables

Landscape variables such as slope, elevation, distance to reserve boundaries, occupied bomas, abandoned bomas, water sources, and infrastructure were obtained using the geographic information systems QGIS (QGIS Development Team 2021) and ArcView (ESRI 2000). Infrastructure included all buildings except bomas as well as fields and fences. Here, we approximate the intensity of human use at the landscape scale, including the presence of livestock, bomas, and infrastructure, based on distance to the reserve boundary. For more details on how landscape variables were measured, see Reid et al. (2003) and Ogutu et al. (2010, 2014).

#### 2.2.7 Upscaling Variables From Sub‐Blocks to Blocks

To relate ungulate diversity and biomass in 1 km‐by‐1 km blocks to the explanatory variables, we averaged all metric variables across all sub‐blocks within each block. We recorded the presence of fire scars, vehicles, and litter, sheep ( *Ovis aries* ) and goats ( *Capra hircus* ), cattle ( *Bos indicus* ), as well as carnivores, including lion ( *Panthera leo* ), spotted hyena ( *Crocuta crocuta* ), cheetah ( *Acinonyx jubatus* ), and leopard ( *Panthera pardus* ) in each block. The summarized data (Appendix S1: Figures S2 and S3a–u; https://doi.org/10.5281/zenodo.16884346: data file “mc_1km.csv”) included all animal observations, human use variables, resource variables, and landscape characteristics. Human use indicators included distances to bomas or infrastructure, as well as the presence of livestock, vehicles, and litter. Resource indicators included rainfall, distances to water, and vegetation attributes. We considered the presence of carnivores as a possible additional explanatory variable while treating slope and elevation as landscape characteristics. All of these variables varied with distance from the Maasai Mara National Reserve in Kenya (Appendix S1: Figures S4–S11).

### 2.3 Data Analysis

#### 2.3.1 Application of Boosting Models

To select the most relevant and predictive variables from our dataset of 26 human use or resource variables, we applied model‐based boosting in R (https://doi.org/10.5281/zenodo.16884346: R code “mc_1km.r”). Boosting is a machine learning method well‐suited for species distribution modeling because it accounts for spatial and temporal effects (Hothorn et al. 2011). It can identify informative predictors from many potentially correlated ones (Mayr et al. 2012) and estimate the contribution of each predictor to ungulate diversity and biomass variation. As strong collinearity makes it difficult to interpret predictions relating to individual variables (Shmueli 2010), we calculated the pairwise correlations between the predictors.

The strongest and most consistent correlations (Spearman's |*ρ*| ≥ 0.75 or Cramer's *V* ≥ 0.75) were found between the different rainfall components across land uses and census years, between distance to the reserve boundary and elevation on pastoral lands, and between sheep and goats and cattle in the reserve across census years (Appendix S1: Figures S4–S15; Appendix S2: Section S4.1). For correlated predictors, stepwise polynomial regression can be misleading due to lack of power (Glantz et al. 2016). Boosting can select between linear and nonlinear terms (Kneib et al. 2009; Smith et al. 2019; Hofner et al. 2011), but this led to overly large models that strain cross‐validation (Mayr, Hofner, et al. 2017). To overcome this, we tuned only the number of iterations in generalized additive models in the R package gamboostLSS version 2.0.6 (Mayr et al. 2012; Hofner, Mayr, and Schmid 2016; Thomas et al. 2018; Hofner et al. 2022).

Because our data are observational, we could not attribute causation to particular predictors. Human use or disturbances such as drought can alter resources, or resources may vary independently of human use or disturbance. To address this, we applied variable coefficients (Hastie and Tibshirani 1990) to fit predictors separately by land use and census year (Appendix S2: Section S3.2). Livestock grazing on pastoral lands reduces resources, while human use in the reserve is low. The census year indicates resource fluctuations between the drought year and the normal rainfall year.

Based on the empirical distributions of ungulate diversity and biomass data, we used negative binomial likelihood and log‐link function to model raw species richness (Appendix S2: Section S3.1). For bias‐adjusted species richness, truncated negative binomial likelihood with a log‐link function was used (Appendix S2: Section S3.1). Diversity measures based on Shannon (order 1), Simpson (order 2), and species evenness (order 10) contained between 18% and 23% ones, but no zeros. To model this distribution, we subtracted unity from the observations, fitted zero‐adjusted gamma models to the reduced values, and added unity back to the predictions (Appendix S2: Section S3.1). Zero‐adjustment was necessary because unadjusted gamma models cannot model the zeros that occurred in biomass estimates of all species (15%–23%), migratory species (52%–62%), or nonmigratory species (18%–26%). These mixed‐distribution models used binomial likelihood and logit link function for the zero part, and gamma likelihood and the log‐link function for the nonzero part (Rigby et al. 2019a; Appendix S2: Section S3.1). They are similar to hurdle models for count data (Mullahy 1986) applied to real numbers with a point probability at zero and simultaneous fitting of both likelihoods (Rigby et al. 2019a). Zero‐inflated models are reparameterized zero‐adjusted submodels (Rigby et al. 2019b).

#### 2.3.2 Model Fitting, Effect Estimation and Variable Selection

We used a component‐wise functional gradient descent boosting algorithm that iteratively selects and fits the most influential predictors (Hothorn et al. 2011) as described in Appendix S2: Section S3.3. Instead of fitting predictors to the response variables, base learners use the gradient (first derivative) of the negative log likelihood as the response (Appendix S2: Section S3.3; Mayr et al. 2012). All predictors enter the models simultaneously as base learners, but only the best‐fitting base learner for the distribution parameter that gives the largest reduction in negative log likelihood is updated in each boosting iteration (Appendix S2: Section S3.3; Thomas et al. 2018). If a base learner is not updated, the corresponding variable is excluded from the set of selected variables (Hofner et al. 2014). The type of base learner and, for spline base learners, the knot locations, the polynomial degree, and the amount of smoothing controlled the effect estimates for the explanatory variables (Appendix A: Spline Regression).

#### 2.3.3 Model Tuning

To prevent overfitting (Mayr, Hofner, et al. 2017), we used cross‐validation to determine the optimal number of boosting iterations (Hofner et al. 2014; Appendix A: Model Tuning). We stopped the algorithm early, shrinking estimates toward zero and regularizing the models (Mayr, Hofner, et al. 2017). This reduces variances, stabilizes predictions, and simplifies models (Mayr and Hofner 2018; Hofner, Mayr, and Schmid 2016). We do not preselect or summarize collinear predictors.

#### 2.3.4 Stability Selection

As a result of stopping the algorithm before convergence, *p* values and confidence intervals for coefficients are currently unavailable for complex boosting models with correlated predictors (Hofner, Kneib, and Hothorn 2016; Mayr, Schmid, et al. 2017; Mayr and Hofner 2018). While *p* values can assess signal versus noise, they do not measure effect size or result importance (Wasserstein and Lazar 2016). To identify consistent predictors, we ranked them based on stability by controlling for the number of predictors with low selection probability (Meinshausen and Bühlmann 2010; Hofner et al. 2015; Appendix A: Stability Selection). Predictors were considered consistent if they were selected for either location or skewness parameters of generalized additive models in at least 70% of random data subsets (Hofner et al. 2015). The selection probability of the scale parameter was considered less relevant, as it is a nuisance distribution parameter that is not necessary for predicting ungulate diversity and biomass (Appendix S2: Section S3.1).

#### 2.3.5 Model Predictions

To improve prediction accuracy, we mapped spatiotemporal trends in ungulate diversity and biomass for each census year using cross‐validated models without prior stability selection (Meinshausen and Bühlmann 2010; Hofner et al. 2015; https://doi.org/10.5281/zenodo.16884346: R code “mc_1km_plots.r,” RData file “mc_1km.RData,” shapefiles in “MMNR_boundary.zip” and “MMNR_border.zip”). We also predicted how these variables varied with distance to the reserve boundary. Other continuous predictors had skewed distributions (Appendix S1: Figures S2 and S3), so they were held constant at their medians, and categorical variables were set to zero.

## 3 Results

### 3.1 Spatiotemporal Trends in Ungulate Diversity and Biomass

**FIGURE A1:**
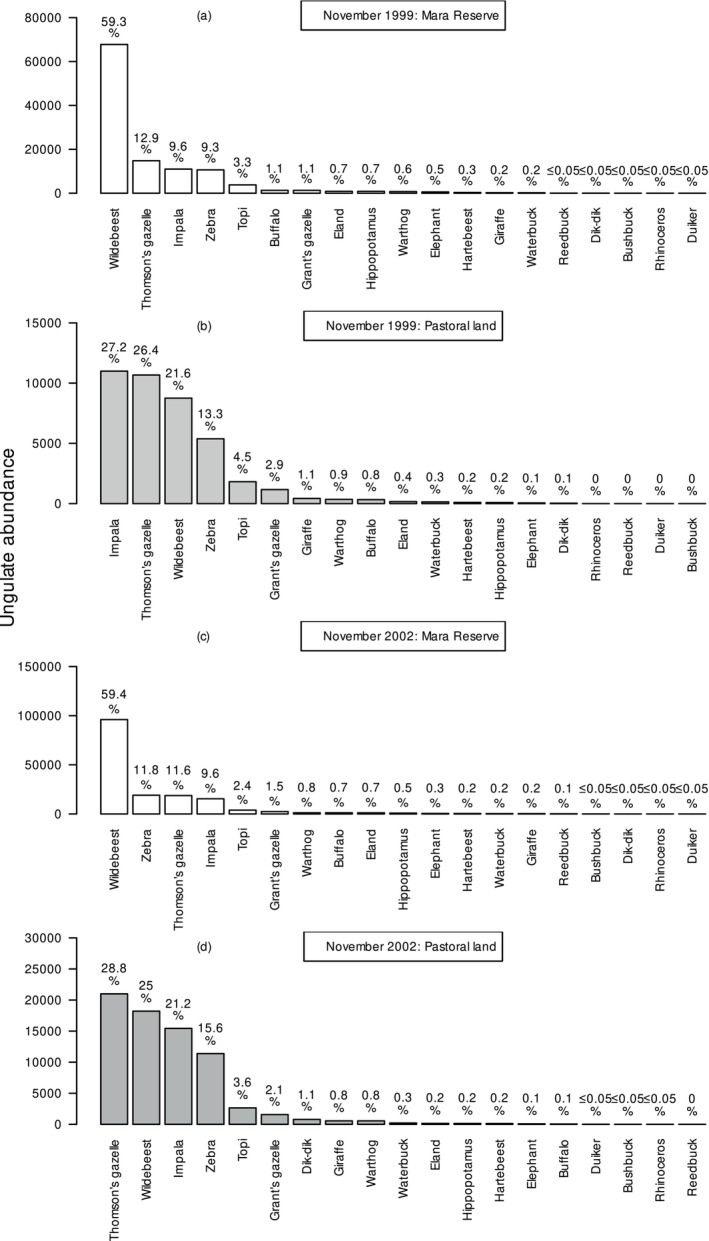
Percentage contribution of each species to the total abundance of savanna ungulates in the Maasai Mara National Reserve (a, c) and adjacent pastoral lands (b, d) in Kenya in November of the 1999 drought year (a, b) and November of the 2002 normal rainfall year (c, d).

**FIGURE A2:**
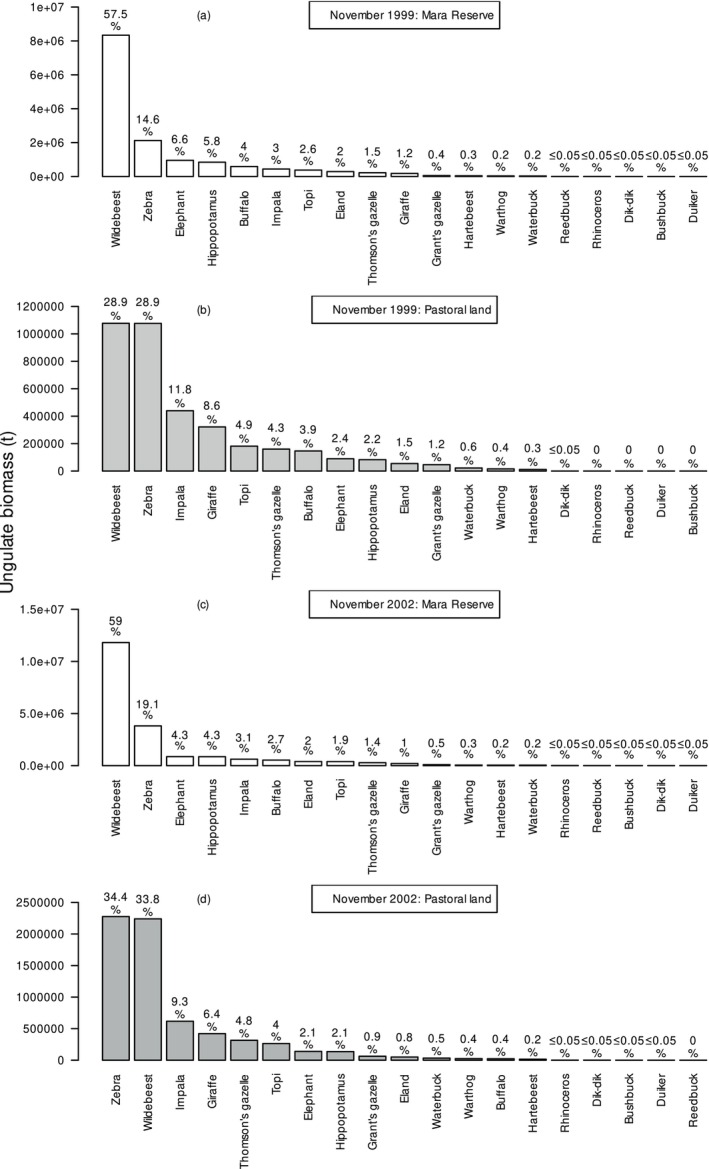
Percentage contribution of each species to total ungulate biomass in November of the 1999 drought year (a, b) and November of the 2002 normal rainfall year (c, d) in the Maasai Mara National Reserve (a, c) and adjacent pastoral lands (b, d) in Kenya.

In the Mara Reserve, ungulate communities had higher species richness and biomass but were less evenly distributed compared with pastoral lands in both census years (Appendix A: Figure A1a–d). Four species were absent from the pastoral lands in the drought year, while only one was observed solely in the reserve in the normal rainfall year (Appendix A: Figure A1a–d). The biomass of ungulates declined overall in both the Mara Reserve and on the pastoral lands in the drought year; although, the decline was less severe within the reserve (Appendix A: Figure A2a–d).

**FIGURE 3:**
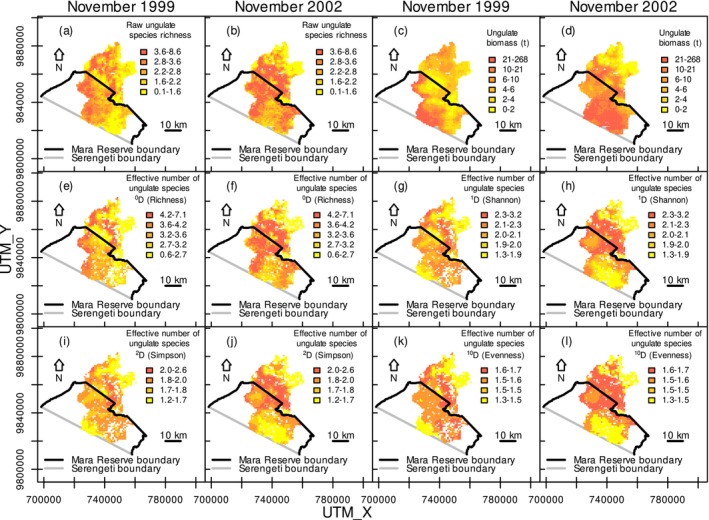
Ungulate diversity expressed as raw species richness (a, b), bias‐adjusted effective number of species based on diversity of orders 0 (richness in e, f), 1 (Shannon in g, h), 2 (Simpson in i, j) and 10 (evenness in k, l), and biomass of savanna ungulates (c, d) in the Maasai Mara National Reserve and adjacent pastoral lands in Kenya in November of the drought year 1999 (a, e, i, c, g, k) and November of the normal rainfall year 2002 (b, f, j, d, h, l).

**FIGURE A3:**
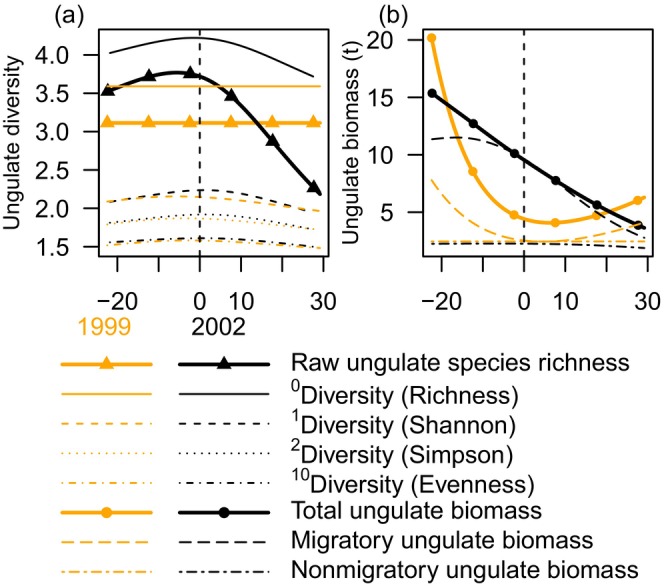
Ungulate diversity (a), expressed as raw species richness (thick continuous lines with triangles in a), bias‐adjusted effective number of species based on diversity orders 0 (species richness, continuous lines in a), 1 (Shannon, dashed lines in a), 2 (Simpson, dotted lines in a) and 10 (species evenness, dashed‐dotted lines in a), and ungulate biomass of all (thick continuous lines with circles in b), migratory (long dashed lines in b), and nonmigratory (double dashed lines in b) savanna ungulates relative to distance from the Maasai Mara National Reserve boundary in Kenya in November of the 1999 drought year (orange lines) and November of the 2002 normal rainfall year (black lines).

Distance to the reserve boundary consistently predicted raw ungulate species richness and biomass, except for nonmigratory species in 1999 (Appendix S3: Tables S1–S4). It also consistently predicted ungulate diversity of different orders in both census years, except for bias‐adjusted species richness in 1999 (Appendix S3: Tables S5–S8). Ungulate diversity peaked within about 10 km of the Mara Reserve boundary in the 2002 normal rainfall year, but drought in 1999 depressed the peak in species richness (Figure 3f,h,j,l; Appendix A: Figure A3a; Appendix S1: Figure S16a–e) variance should decrease with a lower mean (Figure 3a,b,e,f; Rigby et al. 2019a).

Ungulate biomass was dominated by 7 of the 19 species, foremost wildebeest and zebra (Appendix A: Figure A2a–d; Appendix S2: Section S4.2). Within about 20 km of the Mara Reserve boundary, ungulate biomass decreased by up to 50% on both land uses in the drought year compared to the year with normal rainfall (Figure 3c,d; Appendix A: Figure A3b; Appendix S1: Figure S17a). Beyond 20 km from the boundary, biomass increased by up to 31% within the reserve in the drought year compared to the year with normal rainfall (Figure 3c,d; Appendix A: Figure A3b; Appendix S1: Figure S17a). Migratory ungulate biomass trends were similar to all ungulates in 1999 and peaked in the reserve in 2002 (Appendix A: Figure A3b; Appendix S1: Figure S17a,b). Nonmigratory ungulate biomass had a small peak in the Mara Reserve at 7.1 km from the boundary only in 2002 (Appendix A: Figure A3b; Appendix S1: Figure S17c).

### 3.2 Consistent Predictors of Ungulate Diversity and Biomass

Ungulate diversity and biomass were consistently predicted by distance to the reserve boundary, and elevation or occupied bomas on pastoral lands (Appendix S3: Tables S1–S8; Appendix A: Details of Consistent Predictors of Ungulate Diversity and Biomass). Grass color, shrub or tree cover were among the most consistent predictors of ungulate diversity and biomass depending on land use and census year (Appendix S3: Tables S1, S2 and S5–S8; Appendix A: Details of Consistent Predictors of Ungulate Diversity and Biomass). Distance to water and slope were consistent predictors of ungulate species richness in both land uses and both census years, and of ungulate biomass depending on land use and census year (Appendix S3: Tables S1, S2 and S5). Distance to water also consistently predicted ungulate diversity measures after controlling for species evenness in the Mara Reserve in 1999 (Appendix S3: Tables S5–S8).

Other consistent predictors of ungulate diversity and biomass included abandoned bomas, distance to infrastructure, and fire scars depending on land use and census year (Appendix S3: Tables S1, S2 and S5–S8; Appendix A: Details of Consistent Predictors of Ungulate Diversity and Biomass). The presence of sheep and goats, wet season rainfall, grass cover or height, shrub height or color, and tree height or color predicted ungulate diversity more consistently than biomass (Appendix S3: Tables S1, S2 and S5–S8; Appendix A: Details of Consistent Predictors of Ungulate Diversity and Biomass). Tree cover predicted ungulate diversity more consistently in the 1999 drought year than in 2002 (Appendix S3: Tables S1 and S5–S8). Dry season or preceding month's rainfall, grass height, cattle, carnivores, vehicles, and litter were mostly inconsistent predictors of ungulate diversity and biomass (Appendix S3: Tables S1, S2 and S5–S8; Appendix A: Details of Consistent Predictors of Ungulate Diversity and Biomass).

In summary, the human use variables that most consistently predicted ungulate diversity and biomass were distance to the reserve boundary and, for pastoral lands, distance to occupied bomas. Depending on land use and census year, other consistent predictors included distances to abandoned bomas, infrastructure, and fire scars. The presence of sheep and goats was another consistent predictor of diversity. The most consistent predictors of ungulate diversity and biomass among environmental resource variables were grass color, shrub or tree cover, and distance to water. Lastly, slope appeared to be an important landscape characteristic for ungulate diversity.

## 4 Discussion

We expected ungulate diversity to peak at intermediate levels of human use because the high productivity of the Kenyan Mara, driven by fertile soils and seasonal rainfall, can support peak ungulate diversity at intermediate levels of disturbance (Kondoh 2001). Drought may first affect resource‐hungry ungulates (Abraham et al. 2019); yet it could also increase the proportion of species adapted to less productive habitats. Although testing these hypotheses requires experiments and verification through long‐term monitoring, the observed changes in ungulate diversity and biomass in a year of drought compared to a year with normal rainfall provide partial support.

### 4.1 Ungulate Diversity Peaks Along the Boundary of the Maasai Mara National Reserve

The peak in ungulate diversity on the pastoral lands near the Mara Reserve boundary in 2002 is consistent with the predictions of the Intermediate Disturbance Hypothesis (Connell 1978). Elevation, which correlated with distance from the reserve boundary, may have been a confounding predictor of ungulate diversity on pastoral lands. In the Mara Reserve in 2002, rainfall during the wet season and the previous month could have been a confounding predictor of ungulate diversity as it increased from the reserve boundary toward the interior. Some species might also have dispersed from their core habitats in the reserve to less favorable habitats without maintaining viable populations on the pastoral lands, consistent with the mass effect (Shmida and Wilson 1985). This can increase species diversity near habitat boundaries (Kunin 1998).

Our results indicate that occupied bomas, sheep and goats, fire, shrub height and color, rainfall in the wet season, and slope are important predictors of ungulate diversity. Livestock grazing may attract ungulates that avoid areas with tall grass of lower nutritional quality and higher predation risk (Bhola et al. 2012; McNaughton 1984; Hopcraft et al. 2005). Pastoral lands may also be occupied by species displaced from the interior of the reserve by migrating wildebeest and zebra herds (Green et al. 2019; Ogutu et al. 2008). Regrowth after fire attracts more selective grazers (Van de Vijver et al. 1999), while shrub cover and color may benefit mixed feeders and browsers, leading to a more even ungulate community on pastoral lands.

### 4.2 Ungulate Biomass Peaks Inside the Reserve

We expected ungulate biomass to peak at low levels of human use because wildebeest and zebra, which make up the majority of the reserve's ungulate biomass, require substantial resources (Cumming 1982). These species are more vulnerable to disturbance and more mobile than smaller, more resistant ungulate species (Abraham et al. 2019; Kinnaird and O'brien 2012). Although we could not determine whether biomass decreases at zero human use due to burning in the reserve (Veldhuis et al. 2019), peaks in ungulate biomass were observed in the reserve in both the drought and normal rainfall years. The increase in grass color toward the interior of the Mara Reserve may partly explain this peak in the drought year, though it was less pronounced than in the year with normal rainfall. These findings suggest that settlements, livestock (Veldhuis et al. 2019), or fences (Løvschal et al. 2022) also drove ungulates into the reserve. Livestock grazing up to 7 km inside the reserve has probably reduced resources for wild ungulates (Prins 2000; Kimuyu et al. 2017; Young et al. 2018) and displaced them from pastoral areas of high human use on the pastoral lands far from the reserve. Increasing elevation with distance from the boundary may also have influenced trends in ungulate biomass on the pastoral lands.

### 4.3 Ungulate Biomass Depends More Strongly on Resources Than Does Ungulate Diversity

This prediction is supported by the steeper decline in ungulate biomass relative to their diversity in the drought year compared with the normal rainfall year, and on pastoral lands compared with the protected area. As expected, mixed feeders such as Thomson's gazelle and impala increased proportionately in the drought year compared with the normal rainfall year on pastoral lands, and on pastoral lands compared with the reserve in both census years. These species have better access to nutrients and water through browsing than wildebeest and zebra (Kihwele et al. 2020), which dominated the ungulate community in the reserve in the normal rainfall year. The smaller, more resistant ungulate species may contribute to species richness, evenness, and ungulate diversity but less to ungulate biomass (Cumming 1982).

### 4.4 Ungulate Diversity Declines Where Biomass Peaks

The decline in several indicators of ungulate diversity where biomass peaked, such as in the Mara Reserve, suggests that the humped‐back model of species richness and biomass production (Pierce 2014; Grime and Pierce 2012) applies to ungulate species richness and evenness. This was evident from the highly skewed rank‐abundance distribution of individual species for the reserve. Locally high ungulate biomass may reduce ungulate diversity in protected areas through increased interspecific competition or displacement of some species. High ungulate biomass may also affect other species groups (Long et al. 2017; Pringle et al. 2007; Francis et al. 2020).

### 4.5 Ungulate Diversity and Biomass Peaks Partly Shift Toward Less Intense Human Use and More Available Resources During Drought

We expected ungulate species and biomass densities to decrease in low resource areas and instead concentrate in medium‐to‐high resource areas during the drought (Anderson et al. 2012). The higher concentration of ungulate biomass in the interior of the reserve in the drought compared with the normal rainfall year partially supports our prediction. These trends were primarily driven by migratory species such as wildebeest and zebra returning to the Serengeti through the interior of the reserve (Pennycuick 1975; Thirgood et al. 2004; Boone et al. 2006).

### 4.6 Conservation Implications

Our results suggest that intermediate human use in pastoral landscapes can increase ungulate diversity, while protected areas with higher resources are critical for ungulate biomass, especially during droughts. Livestock movements into the reserve could be reduced by effective control mechanisms and incentives for livestock owners to minimize the impact of reduced grazing areas and livestock losses during droughts. Determining the optimal density of bomas and their associated livestock in relation to rainfall requires a more detailed assessment that considers other relevant sources of environmental variation. Our results suggest that reducing the intensity of human use beyond 10 km from the Maasai Mara National Reserve boundary would increase ungulate diversity and biomass.

The expansion of pastoral settlements, livestock, and fences can be limited by creating settlement zones to accommodate growing human populations. Livelihoods can be diversified beyond pastoralism by developing public facilities, sanitation, transport, education, and vocational training, health services, renewable energy, or ecotourism in nature reserves or wildlife conservancies. To control fencing and other land use developments within the ecosystem, conservation‐oriented development approval procedures can be implemented based on far‐sighted spatial planning; more wildlife conservancies may be established.

The Narok County Spatial Plan, the Greater Maasai Mara Ecosystem Management Plan, the Maasai Mara National Reserve Management Plan, and the Narok County Spatial Planning process provide opportunities to implement and develop these strategies in this ecosystem.

### 4.7 Conclusions

Intermediate human use appears to promote higher ungulate diversity with lower biomass compared to low human use. Moderate livestock grazing on pastoral lands may increase the species richness of wild ungulates and lead to a more even distribution of species when tall grass is reduced. Protected areas with low human use provide critical resources for maintaining ungulate biomass, while migratory wildebeest and zebra move into the reserve during droughts. But when locally high biomass is associated with low diversity, it may reduce community stability. Gradients of low‐to‐moderate human use can maintain both ungulate biomass and diversity, while high human use can lead to declines in both. Balancing human use from low‐to‐moderate levels may have contrasting effects on ungulate diversity and biomass, with implications for ecological processes and other species in savannas.

## Author Contributions

**Gundula S. Bartzke:** conceptualization (equal), data curation (equal), formal analysis (lead), investigation (equal), methodology (equal), visualization (lead), writing – original draft (lead), writing – review and editing (equal). **Joseph O. Ogutu:** conceptualization (equal), data curation (equal), formal analysis (supporting), funding acquisition (supporting), investigation (supporting), methodology (equal), project administration (equal), supervision (equal), writing – review and editing (lead). **Hans‐Peter Piepho:** conceptualization (supporting), formal analysis (supporting), funding acquisition (supporting), methodology (supporting), supervision (supporting), writing – review and editing (equal). **Claire Bedelian:** data curation (supporting), investigation (supporting), writing – review and editing (supporting). **Michael E. Rainy:** data curation (supporting), investigation (supporting), writing – review and editing (supporting). **Russel L. Kruska:** data curation (supporting), investigation (supporting), writing – review and editing (supporting). **Jeffrey S. Worden:** data curation (supporting), investigation (supporting), writing – review and editing (supporting). **Kamau Kimani:** data curation (supporting), investigation (supporting), writing – review and editing (supporting). **Michael J. McCartney:** data curation (supporting), investigation (supporting), writing – review and editing (supporting). **Leah Ng'ang'a:** data curation (supporting), investigation (supporting), writing – review and editing (supporting). **Jeniffer Kinoti:** data curation (supporting), investigation (supporting), writing – review and editing (supporting). **Evanson C. Njuguna:** data curation (supporting), investigation (supporting), writing – review and editing (supporting). **Cathleen J. Wilson:** conceptualization (supporting), data curation (supporting), investigation (supporting), supervision (supporting), writing – review and editing (supporting). **Richard Lamprey:** data curation (supporting), investigation (supporting), writing – review and editing (supporting). **Nicholas Thompson Hobbs:** funding acquisition (supporting), writing – review and editing (supporting). **Robin S. Reid:** conceptualization (equal), funding acquisition (lead), investigation (equal), methodology (equal), project administration (equal), supervision (equal), writing – review and editing (equal).

## Conflicts of Interest

The authors declare no conflicts of interest.

## Supporting information


**Appendix S1:** ece371946‐sup‐0001‐AppendixS1.pdf.

**Appendix S2:** ece371946‐sup‐0002‐AppendixS2.pdf.

**Appendix S3:** ece371946‐sup‐0003‐AppendixS3.pdf.

**Appendix S4:** ece371946‐sup‐0004‐AppendixS4.pdf.

## Data Availability

The data and R program codes are provided on https://doi.org/10.5281/zenodo.16884346.

## Appendix A

### Rainfall Components

Total wet season rainfall was strongly correlated with annual rainfall (Spearman's correlation coefficient: 0.94–0.99); we therefore dropped annual rainfall and retained total wet season rainfall as the predictor. Total wet season rainfall may influence juvenile recruitment (Ogutu et al. 2011), reduce births of less water dependent species such as impala (Ogutu et al. 2008), and may alter ungulate diversity and biomass. Total dry season rainfall is important for the survival of water dependent species such as buffalo (Dublin and Ogutu 2015) and wildebeest (Sinclair et al. 1985). Preceding month's rainfall may influence wildebeest migration (Holdo et al. 2009) and thus spatial patterns of ungulate diversity and biomass. In the 1999 drought year, dry season and preceding month's rainfall were higher than in 2002, but wet season rainfall was lower in 1999 (Appendix S1: Figures S2h–k and S3h–j). We captured potential differences in the effects of seasonal and monthly precipitation components on ungulate composition.

### Spline Regression

*P*‐splines are linear combinations of *B*‐splines, slices of polynomial functions, that subtract a roughness penalty from the log likelihood to smooth the regression functions (Eilers and Marx 1996). The penalty depends on the squared second‐order difference of adjacent *B*‐spline coefficients (Mayr et al. 2012) multiplied by a scalar (Eilers and Marx 1996). This scalar is controlled by the spline degrees of freedom (Hofner et al. 2011), which can make splines more or less flexible.

For boosting *P*‐spline base learners, initially weak ones with high bias and low variance are preferred because boosting adapts to higher unknown smoothness (Bühlmann and Yu 2003; Schmid and Hothorn 2008). To reduce the selection bias of variables, they should have the same degrees of freedom (Hofner et al. 2011). However, using both linear and nonlinear base learners with 1 degree of freedom for all metric variables and a common intercept (Kneib et al. 2009; Smith et al. 2019; Hofner et al. 2011) resulted in a model with many similar base learners that reached computational limits in cross‐validation.

To minimize prediction errors, we used 3 degrees of freedom in all *P*‐spline base learners, slightly less than the optimal number for *B*‐splines (Schmid and Hothorn 2008). This approach did not eliminate but reduced the selection bias toward more complex smooth base learners over simple linear factor variable ones (Hofner et al. 2011). To use 3 degrees of freedom for the spatial base learners, we centered their effects around their unpenalized part (Kneib et al. 2009). Otherwise, more than 4 degrees of freedom would have been needed to exceed the order of the differences to construct the penalty term. This resulted in fewer spurious predictions of ungulate diversity and biomass compared to the default settings.

To balance prediction accuracy and interpretability, we used five knots in all smooth base learners. While more knots can model complex patterns such as humps or dips (Ruppert 2002), they can become difficult to interpret without supporting biological hypotheses. Reducing the number of knots to 5 may actually reduce prediction error for less complex relationships, at least for quadratic splines (Ruppert 2002). We evenly spaced knots along the gradient of continuous variables (Eilers and Marx 2010) except for distances to occupied or abandoned bomas and infrastructure. While livestock grazing around bomas is one of the largest potential impacts on wild ungulates, sheep and goat densities became negligible and cattle densities decreased by about 80%–90% beyond 5 km from bomas (Ogutu et al. 2010). Assuming that bomas would be difficult to detect for ungulates beyond 5 km, we placed all five interior knots within 5 km of infrastructure and occupied or abandoned bomas.

### Model Tuning

We used cross‐validation to determine the optimal number of boosting iterations (Hofner et al. 2014). The procedure averaged the negative log likelihood of the models for 25 out‐of‐bag samples not used in model fitting, half the data at a time, for a range of possible boosting iterations (Hofner et al. 2014). The iteration with the lowest average negative log likelihood was chosen as the stopping point for the boosting algorithm (Hofner, Mayr, and Schmid 2016).

### Stability Selection

Stability selection fits models to complementary pairs of random subsets, each containing half the data (Hofner et al. 2015). We used 50 complementary pairs for the ungulate diversity models. For the raw species richness and biomass models of all, migratory, and nonmigratory ungulates, we also used 51 data pairs because a subset contained only a single level of at least one factor variable. Boosting models were fitted to each subset, increasing the number of iterations until 10% of the variables were selected. This controlled for variables with a low probability of selection that may be spuriously selected under unimodal assumptions (Shah and Samworth 2013; Hofner et al. 2015).

### Details of Consistent Predictors of Ungulate Diversity and Biomass

Distance to the reserve boundary was, except for species richness and biomass of migratory species in 1999, the most consistent predictor of ungulate diversity and biomass (Appendix S3: Tables S1–S8). Elevation was the most consistent predictor of ungulate diversity and biomass on the pastoral lands, while slope was the most consistent predictor of ungulate species richness on both land uses (Appendix S3: Tables S1, S2 and S5–S8). Occupied bomas were, with the exception of ungulate biomass in 1999, one of the most consistent predictors of ungulate diversity and biomass on the pastoral lands (Appendix S3: Tables S1, S2 and S5–S8). Abandoned bomas or the presence of sheep and goats in both census years and the preceding month's rainfall in 2002 were consistent predictors of ungulate species richness on the pastoral lands (Appendix S3: Tables S1 and S5). Distance to infrastructure was a consistent predictor of the diversity measures accounting for species evenness in the Mara Reserve in both census years, ungulate biomass, and raw species richness on the pastoral lands in 2002, and ungulate biomass in the reserve in 1999 (Appendix S3: Tables S1, S2 and S5–S8). Fire was a consistent predictor of ungulate species richness on the pastoral lands in 2002 (Appendix S3: Tables S1 and S5).

Grass color was the most consistent predictor of ungulate biomass in the Mara Reserve in both census years (Appendix S3: Table S2). Grass color and, except for bias‐adjusted species richness in 1999, distance to water were the most consistent predictors of ungulate diversity in the reserve, except for Simpson diversity and species evenness in 1999 (Appendix S3: Tables S1 and S5–S8). Shrub cover was, except for raw species richness in 1999, a consistent predictor of ungulate diversity in the reserve in both census years and of ungulate biomass in the reserve in 1999 (Appendix S3: Tables S1, S2 and S5–S8). Shrub height was a consistent predictor of ungulate diversity accounting for species evenness on the pastoral lands in 1999 and for Shannon and Simpson ungulate diversity in the reserve in 2002 (Appendix S3: Tables S5–S8). Shrub color was a consistent predictor of the bias‐adjusted diversity measures on the pastoral lands in 2002 (Appendix S3: Tables S6–S8). Tree cover was a consistent predictor of ungulate diversity for both land uses and ungulate biomass on pastoral lands in 1999 (Appendix S3: Tables S1, S2 and S5–S8). Tree height was a consistent predictor of ungulate diversity on the pastoral lands and ungulate biomass in the Mara Reserve in 2002, while tree color was, except for the Simpson diversity and species evenness, a consistent predictor of ungulate diversity on both land uses in 1999 (Appendix S3: Tables S1, S5 and S6). Grass cover was a consistent predictor of ungulate species richness in the reserve in 2002 (Appendix S3: Tables S1 and S5).

Wet season rainfall was a consistent predictor of species richness in the Mara Reserve in 1999 and of diversity measures accounting for species evenness on the pastoral lands in 2002 (Appendix S3: Tables S1 and S5–S8). Dry season rainfall, grass height, cattle, carnivores, vehicles, and litter were mostly inconsistent predictors of ungulate diversity and biomass (Appendix S3: Tables S1, S2 and S5–S8).
