# Supplementary material for: Comparative Ungulate Diversity and Biomass Change With Human Use and Drought: Implications for Community Stability and Protected Area Prioritization in African Savannas
Source: Ecol Evol. 2025 Aug 28;15(9):e71946. doi: 10.1002/ece3.71946 (PMC12391912; doi:10.1002/ece3.71946)
Supplement: Supplementary file 1 — Appendix S1: ece371946‐sup‐0001‐AppendixS1.pdf. [file ECE3-15-e71946-s003.pdf]

Appendix S1

Comparative ungulate diversity and biomass change with human use and drought: implications for community stability and protected area prioritization in African savannas

Ecology and Evolution

Gundula S. Bartzke, Joseph O. Ogutu, Hans-Peter Piepho, Claire Bedelian, Michael E. Rainy, Russel L. Kruska, Jeffrey S. Worden, Kamau Kimani, Michael J. McCartney, Leah Ng'ang'a, Jeniffer Kinoti, Evanson C. Njuguna, Cathleen J. Wilson, Richard Lamprey, N. Thompson Hobbs, Robin S. Reid

List of Figures

Figure S1 3
Figure S2 4
Figure S3 5
Figure S4 6
Figure S5 7
Figure S6 8
Figure S7 9
Figure S8 10
Figure S9 11
Figure S10 12
Figure S11 13
Figure S12 14
Figure S13 14
Figure S14 15
Figure S15 15

Figure S16 16
Figure S17 17

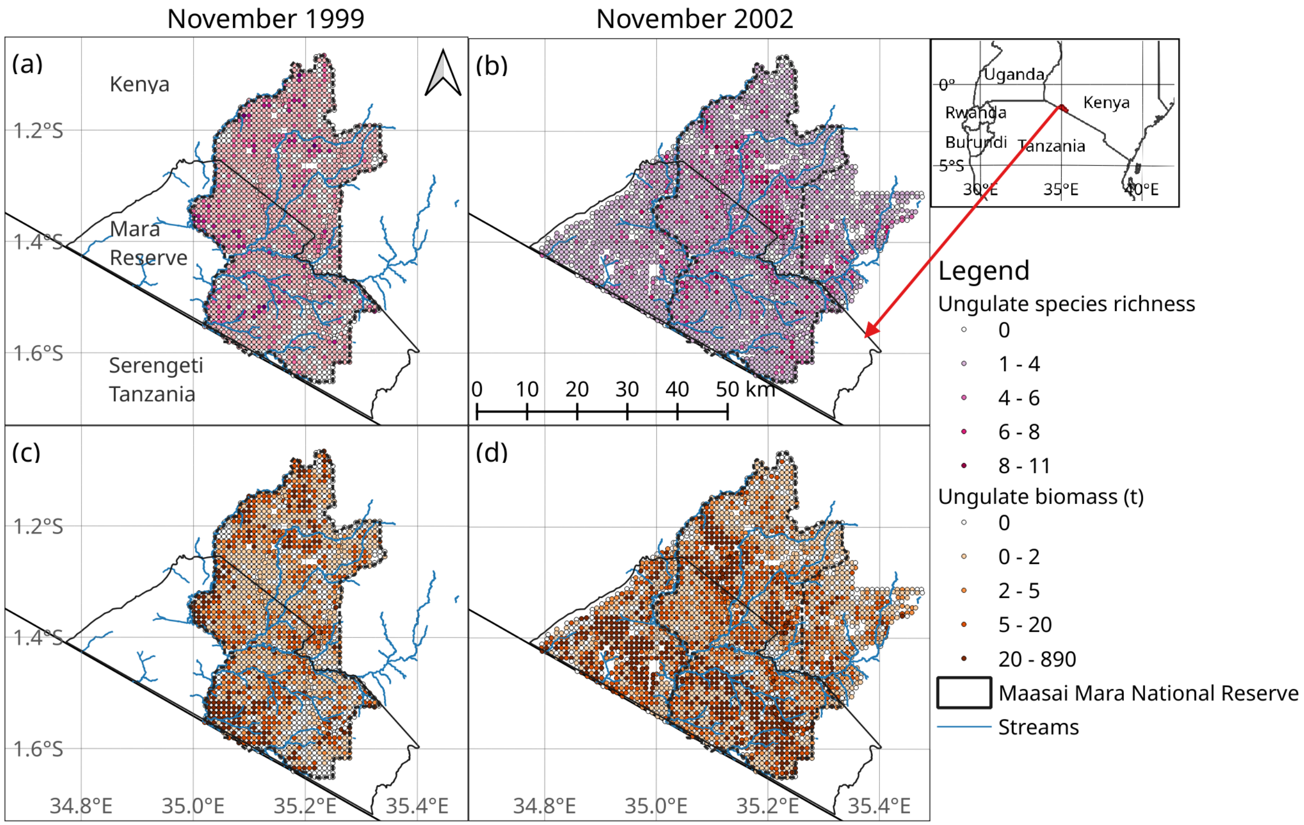

Figure S1: Spatial distribution of raw species richness (a, b: purple circles) and biomass (c, d: orange circles) of savanna ungulates and major streams (blue lines) in the Maasai Mara National Reserve (black polygon) and adjacent pastoral lands in Kenya in November of the 1999 drought year 1999 and November of the 2002 normal rainfall year.

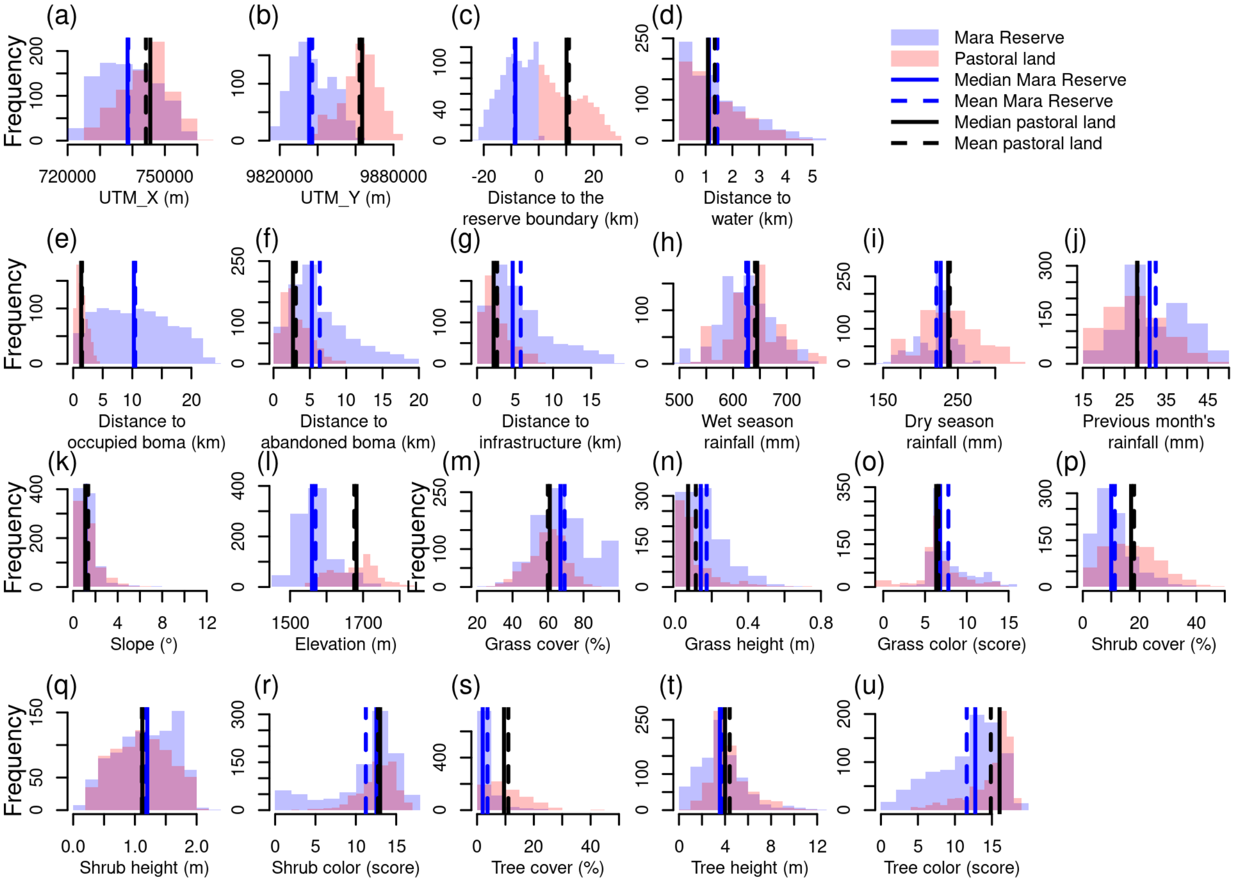

Figure S2: Frequency distributions, medians (continuous lines), and means (dashed lines) of the continuous explanatory variables in all census blocks used to model ungulate diversity and biomass in the Maasai Mara National Reserve (blue bars and blue lines) and adjacent pastoral lands (orange bars and black lines) in Kenya in November of the 1999 drought year.

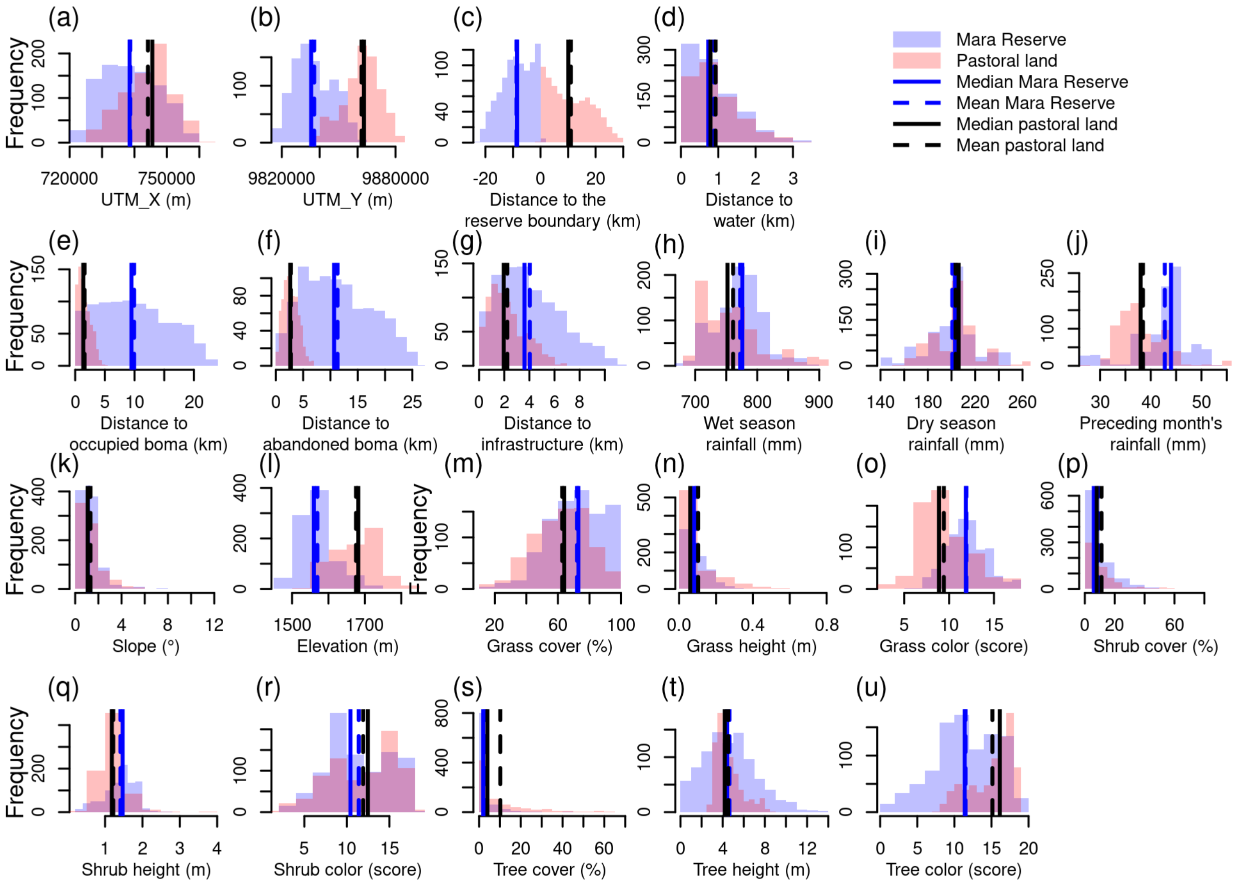

Figure S3: Frequency distributions, medians (continuous lines), and means (dashed lines) of the continuous explanatory variables in all census blocks used to model ungulate diversity and biomass in the Maasai Mara National Reserve (blue bars and blue lines) and adjacent pastoral lands (orange bars and black lines) in Kenya in November of the 2002 normal rainfall year.

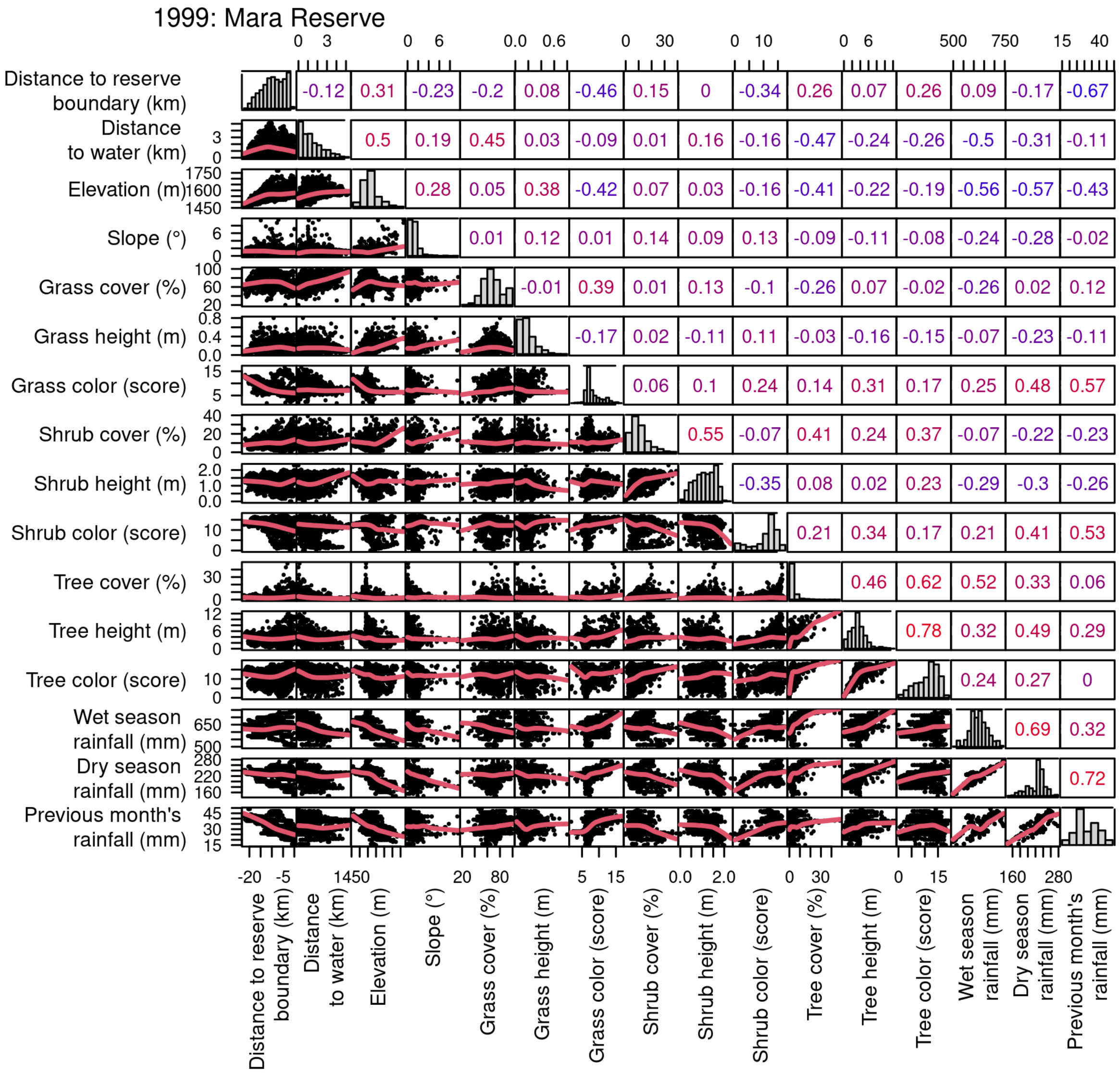

Figure S4: Relationships (red lines) and Spearman's correlation (blue for negative and red for positive coefficients) between the continuous explanatory variables in all census blocks used to model ungulate diversity and biomass in the Maasai Mara National Reserve in southwestern Kenya in November of the 1999 drought year.

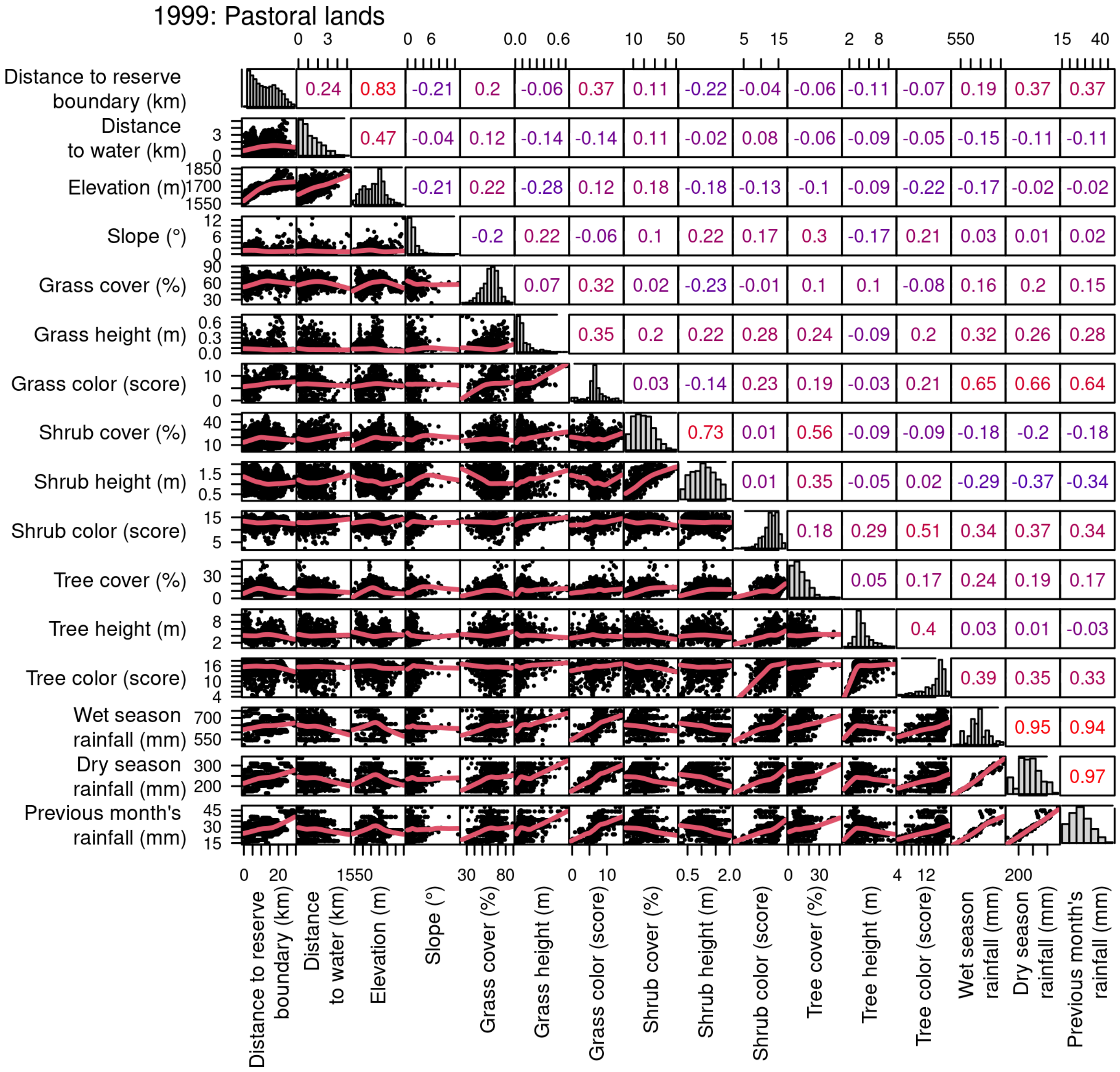

Figure S5: Relationships (red lines) and Spearman's correlation (blue for negative and red for positive coefficients) between the continuous explanatory variables in all census blocks used to model ungulate diversity and biomass on the pastoral lands in the Mara ecosystem in southwestern Kenya in November of the 1999 drought year.

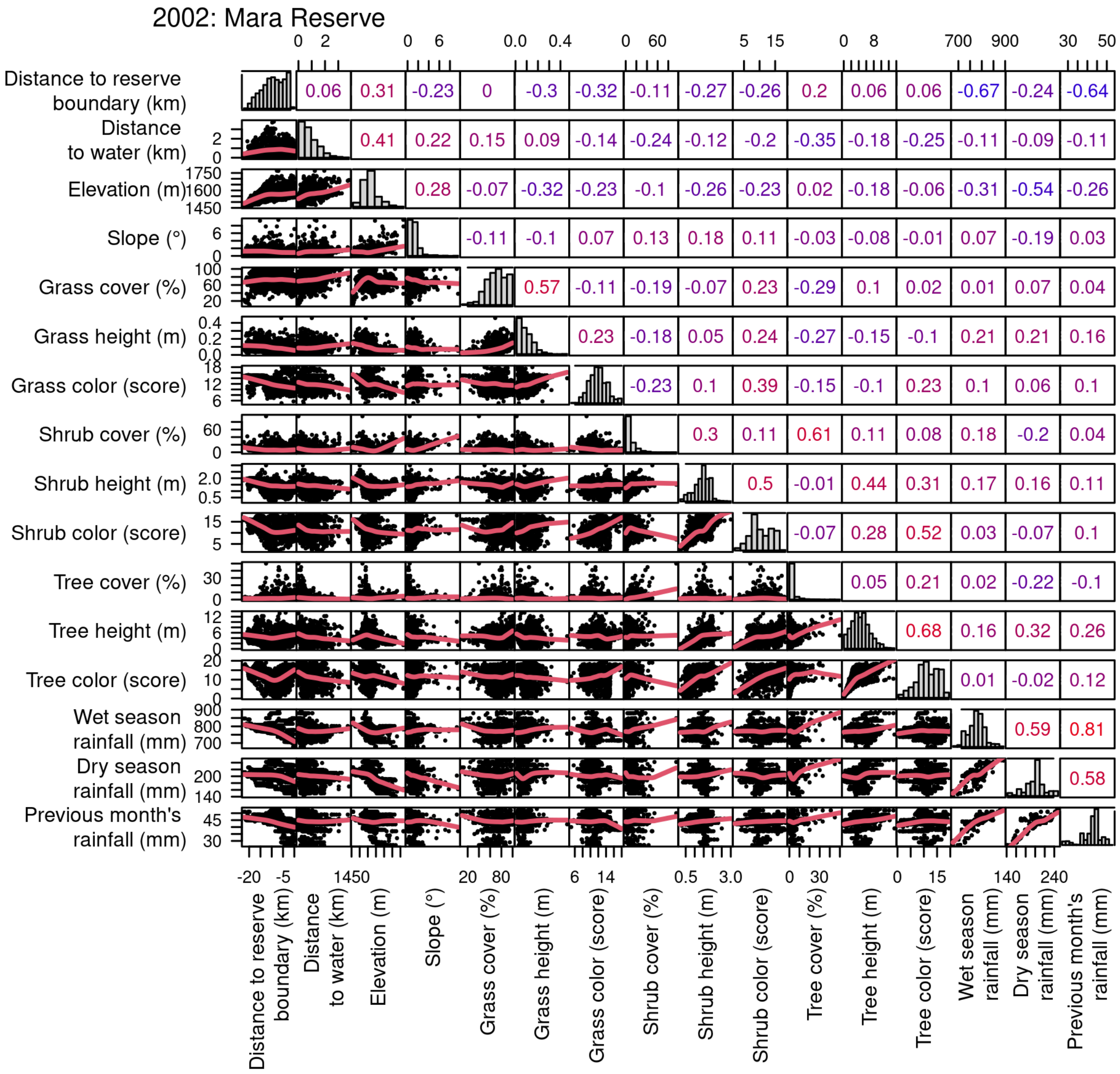

Figure S6: Relationships (red lines) and Spearman's correlation (blue for negative and red for positive coefficients) between the continuous explanatory variables in all census blocks used to model ungulate diversity and biomass in the Maasai Mara National Reserve in southwestern Kenya in November of the normal rainfall year of 1999.

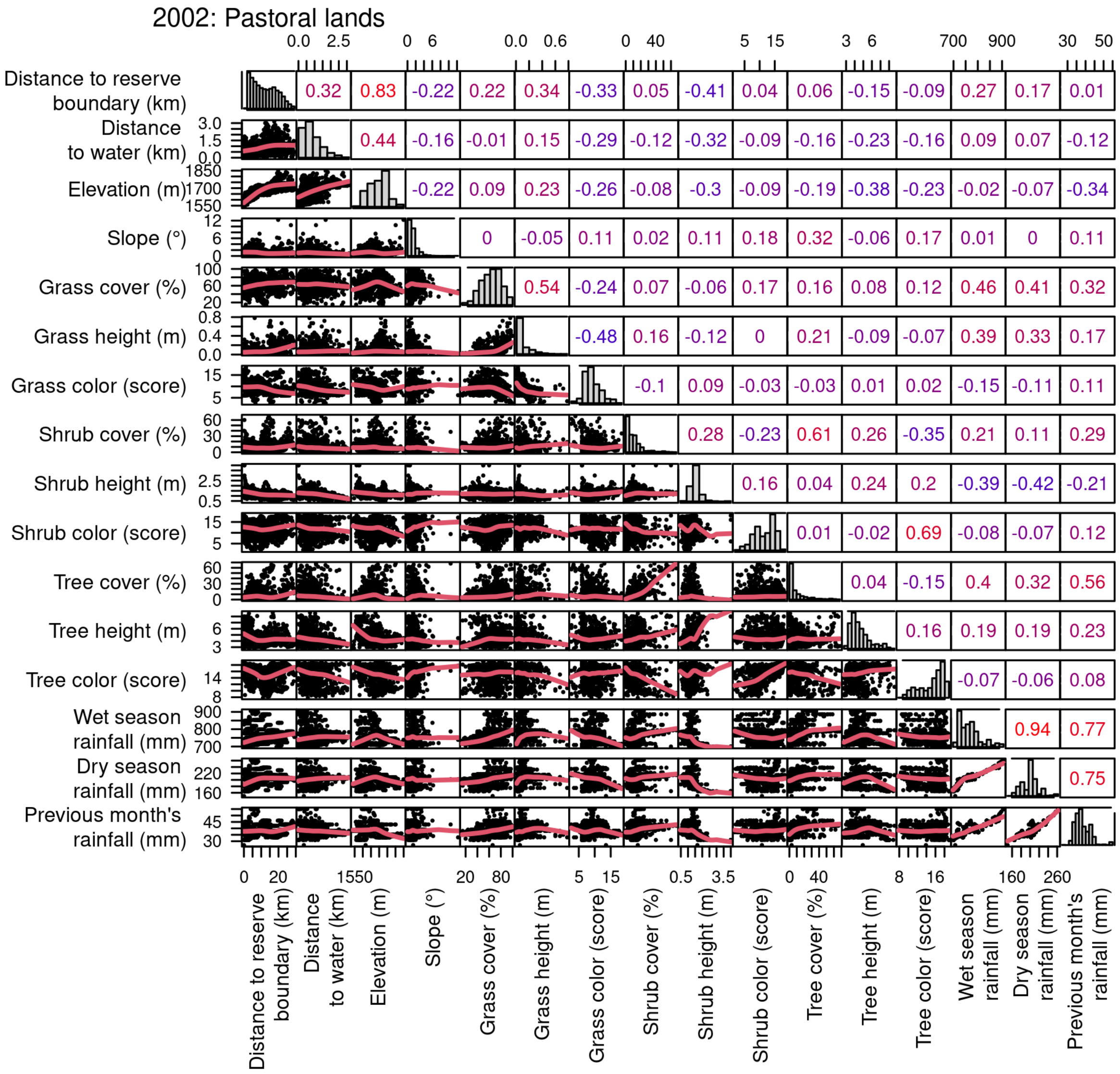

Figure S7: Relationships (red lines) and Spearman's correlation (blue for negative and red for positive coefficients) between the continuous explanatory variables in all census blocks used to model ungulate diversity and biomass on the pastoral lands in the Mara ecosystem in southwestern Kenya in November of the 2002 normal rainfall year.

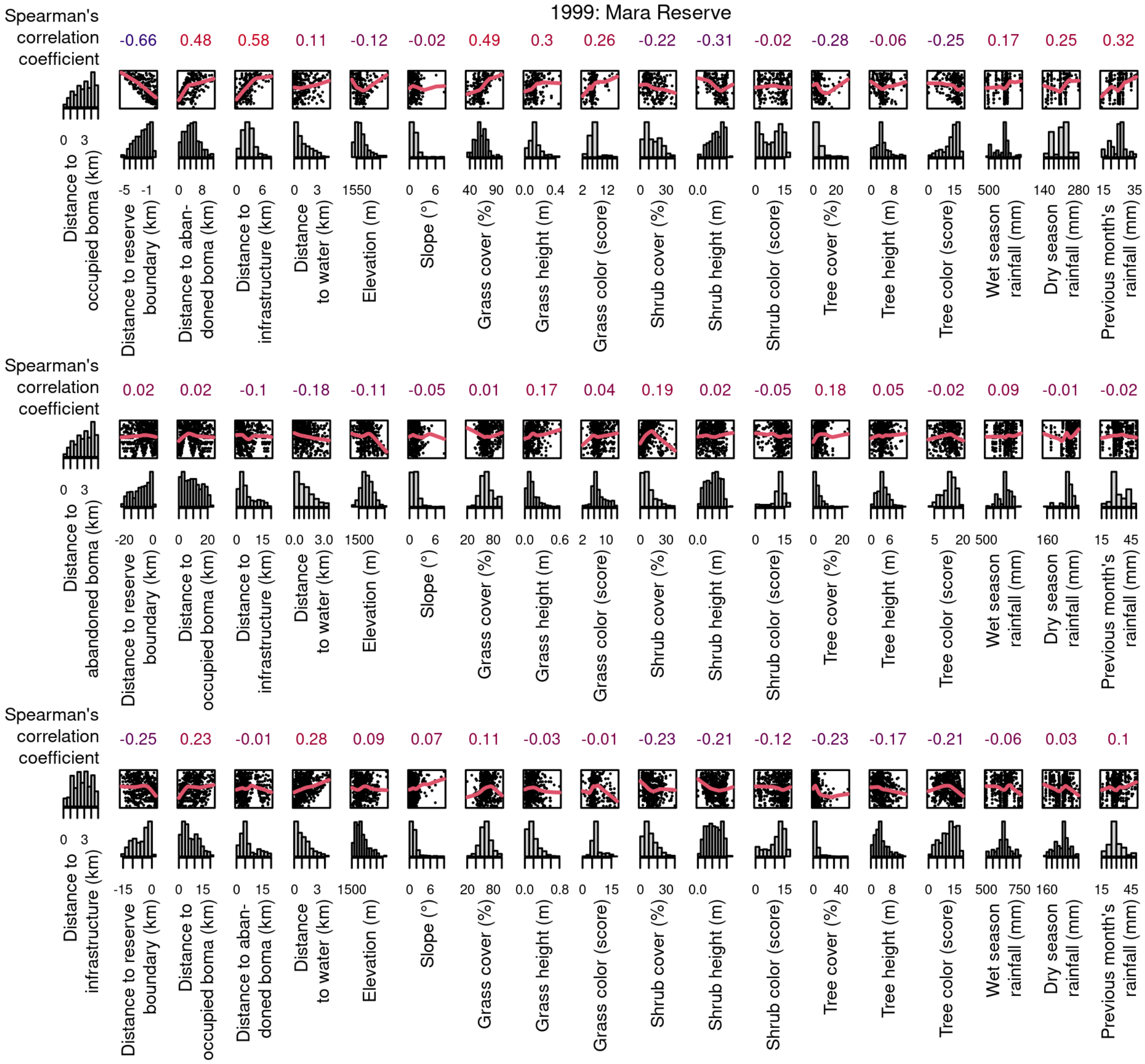

Figure S8: Relationships (red lines) and Spearman's correlation (blue for negative and red for positive coefficients) between distances within 5 km of occupied bomas, abandoned bomas or infrastructure and the other continuous explanatory variables in all census blocks used to model ungulate diversity and biomass in the Maasai Mara National Reserve in southwestern Kenya in November of the 1999 drought year.

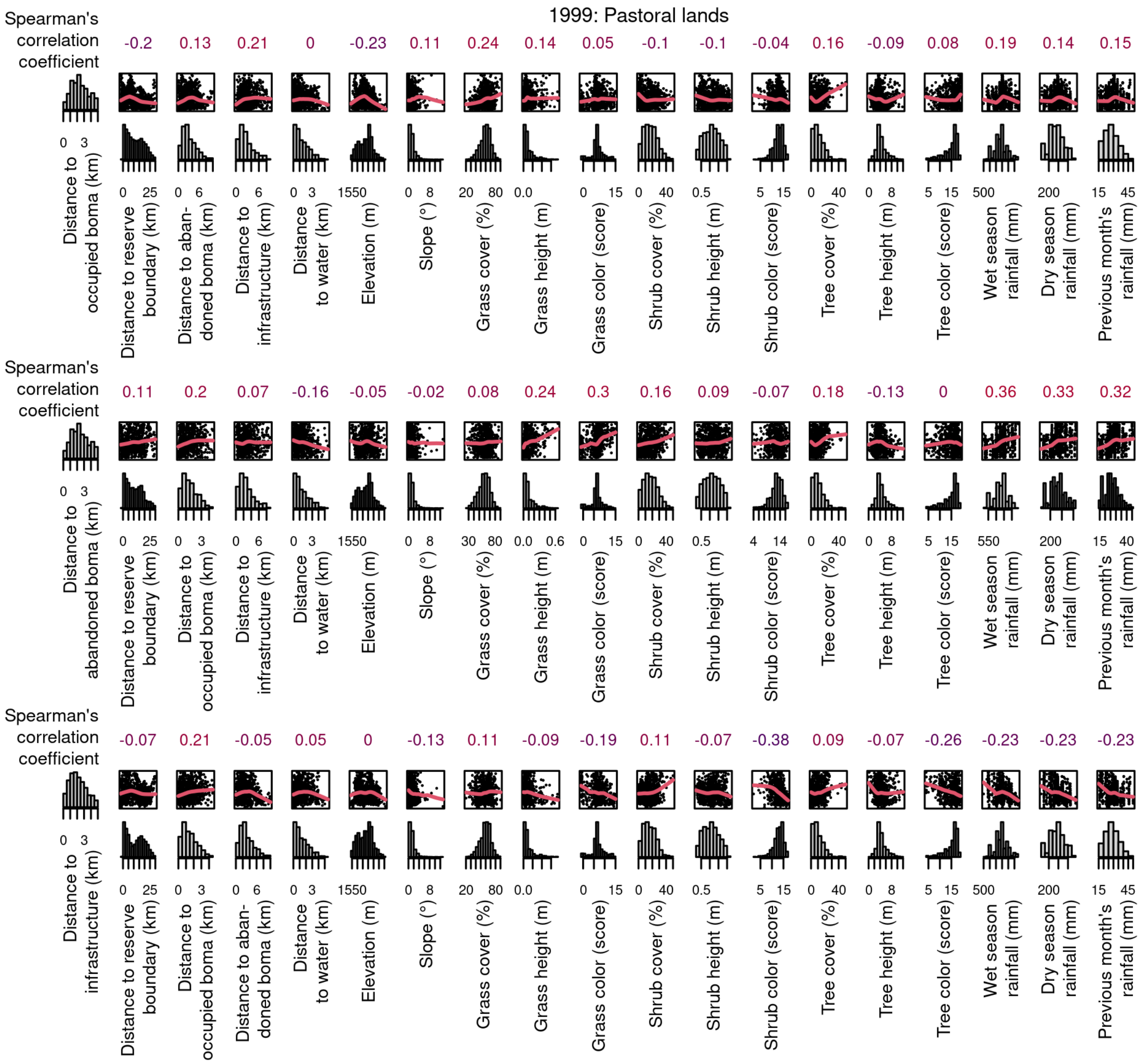

Figure S9: Relationships (red lines) and Spearman's correlation (blue for negative and red for positive coefficients) between distances within 5 km of occupied bomas, abandoned bomas or infrastructure and the other continuous explanatory variables in all census blocks used to model ungulate diversity and biomass on the pastoral lands in the Mara ecosystem in southwestern Kenya in November of the 1999 drought year.

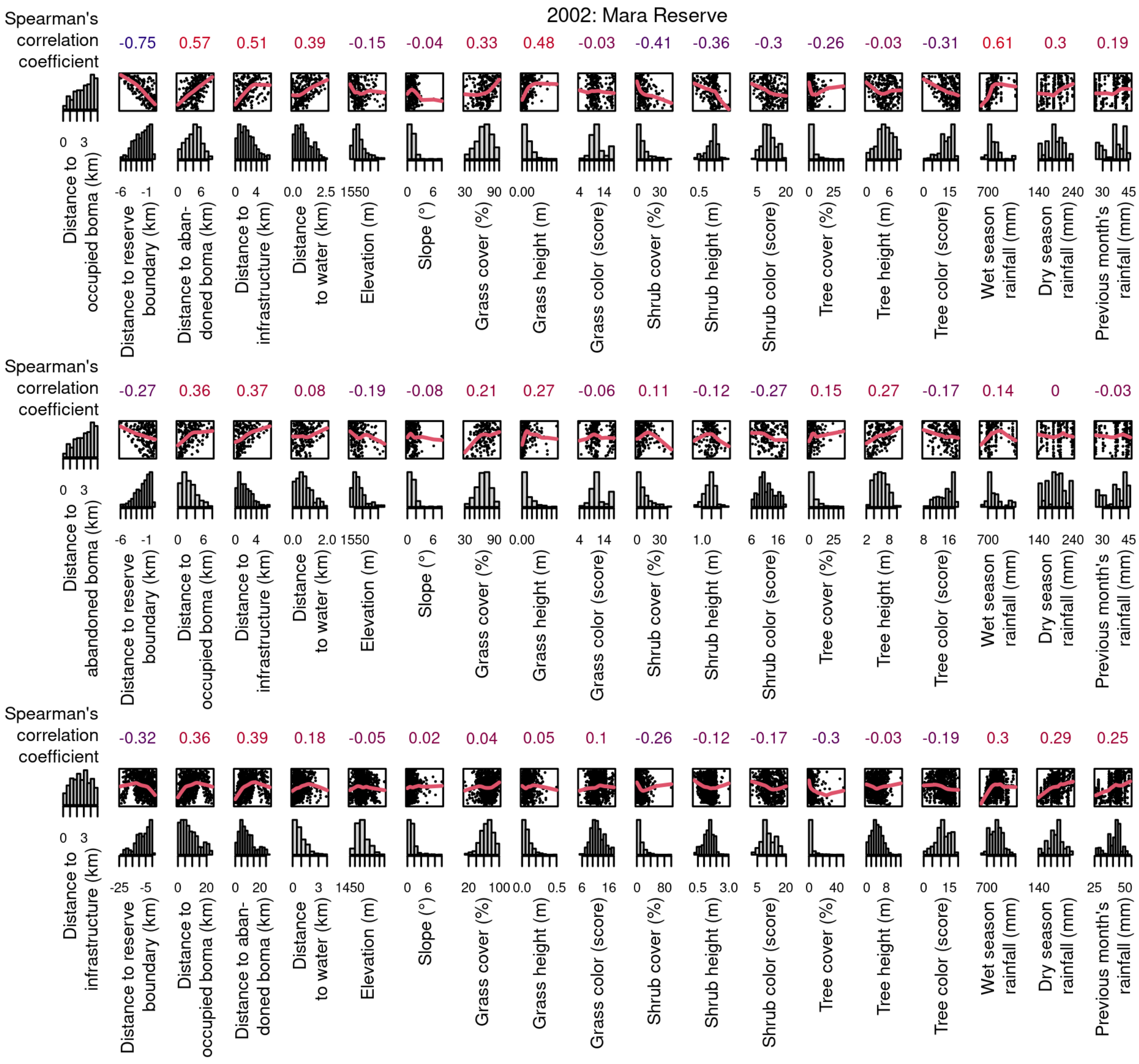

Figure S10: Relationships (red lines) and Spearman's correlation (blue for negative and red for positive coefficients) between distances within 5 km of occupied bomas, abandoned bomas or infrastructure and the other continuous explanatory variables in all census blocks used to model ungulate diversity and biomass in the Maasai Mara National Reserve in southwestern Kenya in November of the 2002 normal rainfall year.

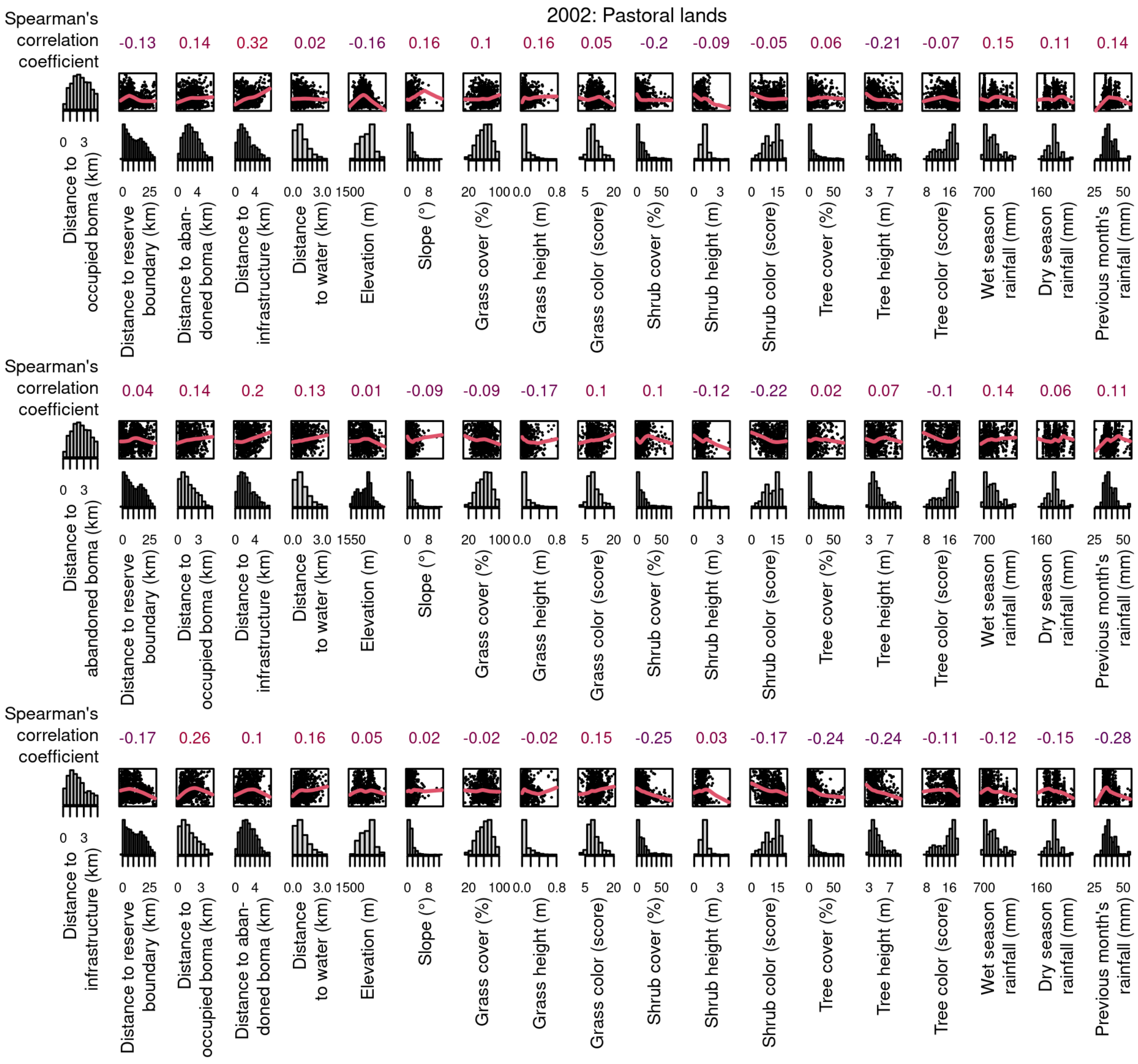

Figure S11: Relationships (red lines) and Spearman's correlation (blue for negative and red for positive coefficients) between distances within 5 km of occupied bomas, abandoned bomas or infrastructure and the other continuous explanatory variables in all census blocks used to model ungulate diversity and biomass on the pastoral lands in the Mara ecosystem in southwestern Kenya in November of the 2002 normal rainfall year.

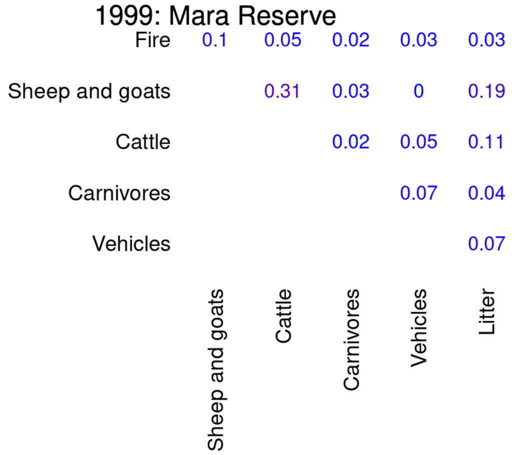

Figure S12: Associations (Cramer's V: blue for low and red for high coefficients) between the categorical explanatory variables in all census blocks used to model ungulate diversity and biomass in the Maasai Mara National Reserve in southwestern Kenya in November of the 1999 drought year.

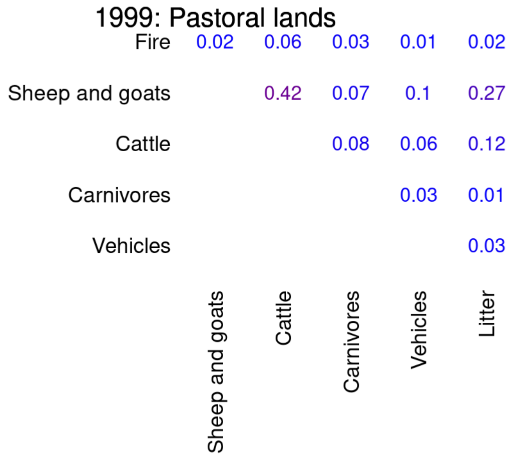

Figure S13: Associations (Cramer's V: blue for low and red for high coefficients) between the categorical explanatory variables in all census blocks used to model ungulate diversity and biomass on the pastoral lands in the Mara ecosystem in southwestern Kenya in November of the 1999 drought year.

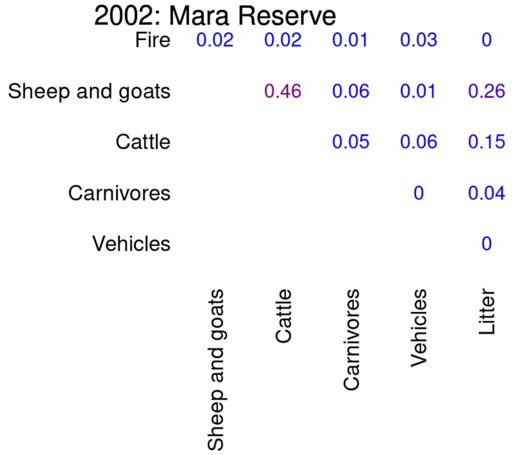

Figure S14: Associations (Cramer's V: blue for low and red for high coefficients) between the categorical explanatory variables in all census blocks used to model ungulate diversity and biomass in the Maasai Mara National Reserve in southwestern Kenya in November of the normal rainfall year of 1999.

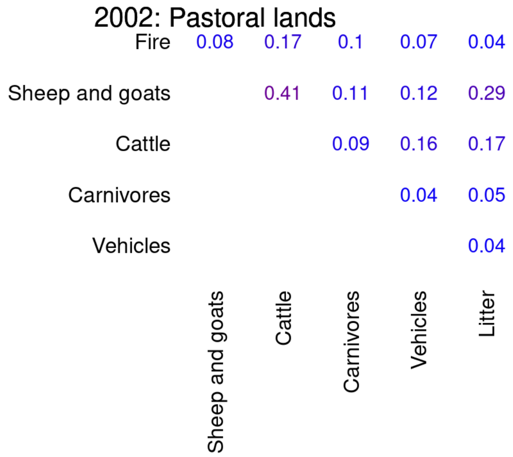

Figure S15: Associations (Cramer's V: blue for low and red for high coefficients) between the categorical explanatory variables in all census blocks used to model ungulate diversity and biomass on the pastoral lands in the Mara ecosystem in southwestern Kenya in November of the 2002 normal rainfall year.

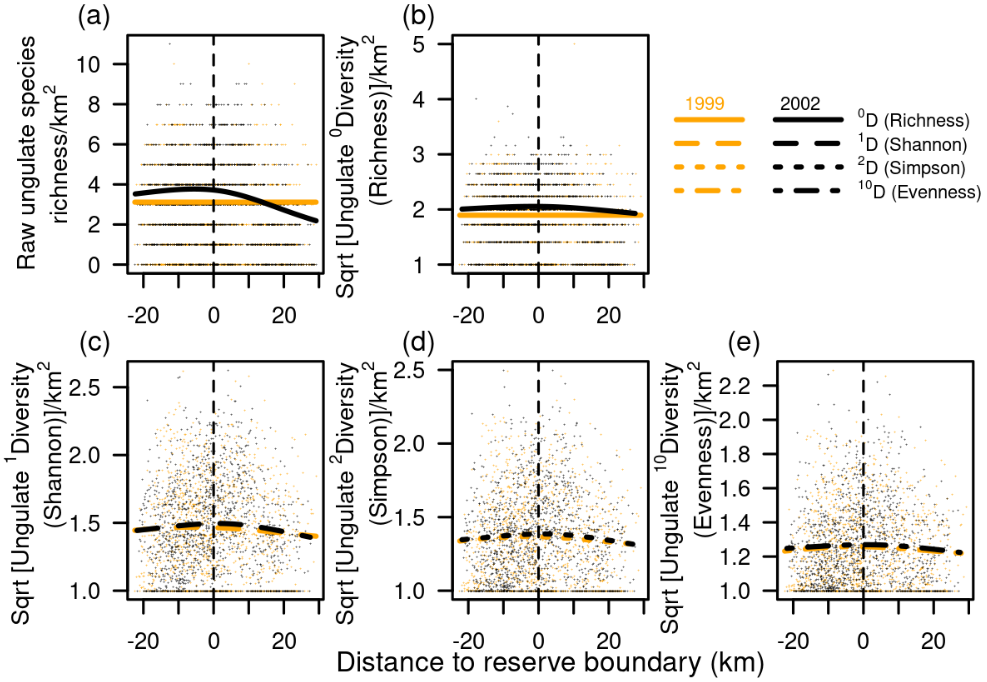

Figure S16: Trends in ungulate diversity expressed as raw species richness (a) or the square root of the bias-adjusted effective number of species of savanna ungulates based on diversity orders 0 (richness; continuous lines in b), 1 (Shannon; dashed lines in c), 2 (Simpson; dotted lines in d) or 10 (species evenness; dotdashed lines in e) relative to the Maasai Mara National Reserve boundary in Kenya in November of the 1999 drought year 1999 (orange lines for predictions in and orange pluses for observations in a-e) and in November of the 2002 normal rainfall year (black lines for predictions and black pluses for observations in a-e).

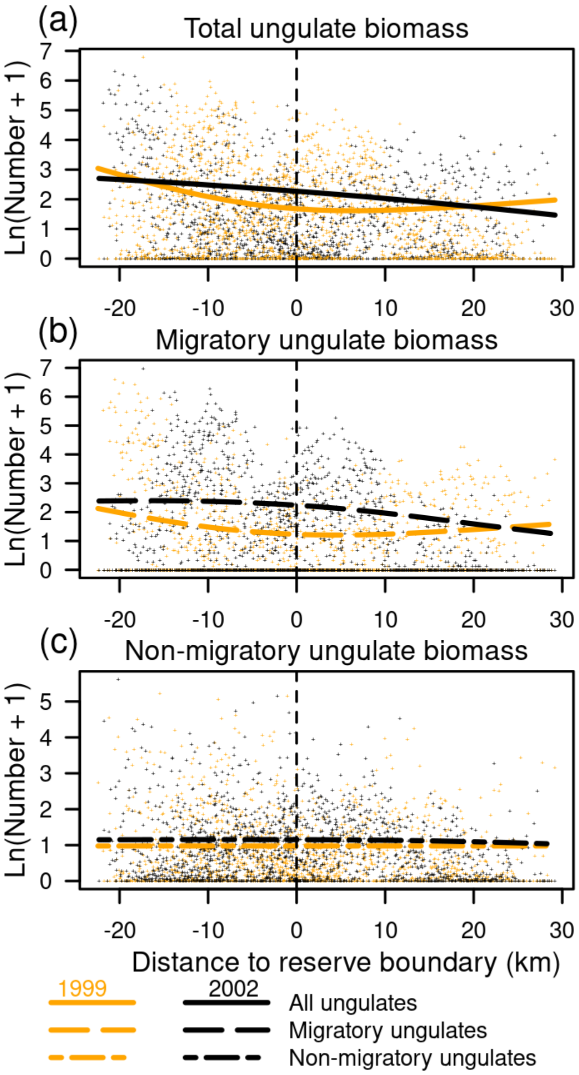

Figure S17: Log-transformed biomass of all (continuous lines in a), migratory (dashed lines in b) or non-migratory (dotted lines in c) savanna ungulates relative to distance from the Maasai Mara National Reserve boundary in Kenya in November of the 1999 drought year 1999 (orange lines for predictions and orange pluses for observations) and November of the 2002 normal rainfall year (black lines for predictions and black pluses for observations).
