# Supplementary material for: Comparative Ungulate Diversity and Biomass Change With Human Use and Drought: Implications for Community Stability and Protected Area Prioritization in African Savannas
Source: Ecol Evol. 2025 Aug 28;15(9):e71946. doi: 10.1002/ece3.71946 (PMC12391912; doi:10.1002/ece3.71946)
Supplement: Supplementary file 2 — Appendix S2: ece371946‐sup‐0002‐AppendixS2.pdf. [file ECE3-15-e71946-s004.pdf]

Appendix S2

Comparative ungulate diversity and biomass change with human use and drought: implications for community stability and protected area prioritization in African savannas

Ecology and Evolution

Gundula S. Bartzke, Joseph O. Ogutu, Hans-Peter Piepho, Claire Bedelian, Michael E. Rainy, Russel L. Kruska, Jeffrey S. Worden, Kamau Kimani, Michael J. McCartney, Leah Ng'ang'a, Jeniffer Kinoti, Evanson C. Njuguna, Cathleen J. Wilson, Richard Lamprey, N. Thompson Hobbs, Robin S. Reid

Contents

Section S1: Kriging method used to impute missing vegetation observations **2**

Section S2: Estimating the diversity of savanna ungulates **5**

Section S3: Modeling ungulate diversity and biomass **8**

Section S3.1: Boosting models 8

Section S3.2: Decomposing regression functions 13

Section S3.3: Fitting boosting models 14

Section S4: Extended results **19**

Section S4.1: Correlations among predictors 19

Section S4.2: Species contributions to the biomass and abundance of savanna ungulates 20

S1: Kriging method used to impute missing vegetation observations

To impute vegetation cover, height and color for unsampled sub-blocks, we used automated kriging in the R package automap version 1.0.16 (Hiemstra et al., 2008). Kriging assumes a constant stationary mean $\hat{\mathrm{x}}(\dot{s})$ and minimizes the estimation variance

$$\sigma_\epsilon^2 = (\dot{\mathrm{x}}(\dot{s}_0) - \hat{\mathrm{x}}(\dot{s}_0))^2, \tag{1}$$

where $\dot{s}_0$ is a prediction location (Bivand et al., 2008c). Predictions for new locations were made from data at sample locations weighted by their variances and spatial covariances (Bivand et al., 2008c).

The prediction for a sub-block with missing observations is given by

$$\hat{\mathrm{x}}(\dot{s}_0) = \mathbf{v}'\mathbf{V}^{-1}\dot{\mathbf{x}}(\dot{\mathbf{s}}), \tag{2}$$

where $\dot{\mathbf{x}}(\dot{\mathbf{s}})$ represents vegetation cover, height or colour at sample locations $\dot{\mathbf{s}}$ and $\mathbf{v}'\mathbf{V}^{-1}$ are kriging weights (Bivand et al., 2008c). The weights are computed from $\mathbf{v}$, which contains variances and spatial covariances between sampled and prediction sub-blocks, multiplied by the matrix $\mathbf{V}$, which contains variances and spatial covariances between sampled sub-blocks (Bivand et al., 2008c). Sample data farther from a prediction location contribute less to the prediction than data closer to the prediction location due to decreasing covariances with distance.

To compute kriging weights, variogram models are used to estimate variances and spatial covariances (Bivand et al., 2008a). The models are fitted to sample variograms that

estimate half of the variances or covariances between observations at N_h pairs of sample locations

$$\hat{\tau}(\tilde{h}_{\jmath}) = \frac{1}{2N_h}\sum_{\acute{\imath}=1}^{N_h}(\mathrm{x}(\acute{s}_{\acute{\imath}}) - \mathrm{x}(\acute{s}_{\acute{\imath}} + h))^2,\ \forall h \in \tilde{h}_{\jmath} \tag{3}$$

separated by distances $\tilde{h}_{\jmath}$ (Bivand et al., 2008a). The relationship between distance and semivariance can be estimated using several variogram models, including spherical, exponential, Gaussian and Stein's models. The R package automap uses the autoKrige function to automatically fit these models (Hiemstra et al., 2008). Initial values for the variogram model parameters are estimated from the observed data before selecting the final variogram model with the smallest residual sum of squares for the sample variogram (Hiemstra et al., 2008).

The variogram-based estimates of variance and covariance are used to impute values for sub-blocks with missing observations using the kriging procedure. The model-based estimate of variance is twice the semivariance at a separation distance called range, where the semivariance is approximately half the variance (Bivand et al., 2008b). For distances below the range, the model-based estimate of the covariance is determined as the difference between the variance and twice the semivariance. This information allows us to complete the observed vegetation data using kriging.

References

Bivand, R. S., E. J. Pebesma, and V. Gómez-Rubio (2008a). 8.4 Estimating spatial correlation: the variogram. In *Applied Spatial Data Analysis with R*, pp. 195–196. New York, NY: USA: Springer.

Bivand, R. S., E. J. Pebesma, and V. Gómez-Rubio (2008b). 8.4.3 Variogram modelling. In *Applied Spatial Data Analysis with R*, Chapter 8.4 Estimating spatial correlation: The variogram, pp. 201–205. New York, NY, USA: Springer.

Bivand, R. S., E. J. Pebesma, and V. Gómez-Rubio (2008c). 8.5 Spatial prediction. In *Applied Spatial Data Analysis with R*, pp. 209. New York, NY, USA: Springer.

Hiemstra, P. H., E. J. Pebesma, C. J. W. Twenhöfel, and G. B. M. Heuvelink (2008). Real-time automatic interpolation of ambient gamma dose rates from the dutch radioactivity monitoring network. *Computers & Geosciences 35*(8), 1711–1721.

S2: Estimating the diversity of savanna ungulates

The bias-adjusted ungulate species richness for blocks with at least one observed species, ${}^0D_i$ for all $i \in \{i|S_i \geq 1\}$, is the jackknife estimator (Burnham and Overton, 1979). All ungulate species are equally weighted in this estimatior.

To account for evenness in the estimation of ungulate diversity, we derived

$$ {}^1D_i = e^{H_i}, \quad \text{where } H_i = -\sum_u \tilde{p}_{ui} \times ln(\tilde{p}_{ui}) \; \forall i \in \{i|S_i \geq 1\} \tag{1} $$

using Shannon's entropy (Shannon, 1948; Hill, 1973), which weights species u by their relative abundance estimates $\tilde{p}_{ui}$ (Pellens and Grandcolas, 2016) and e is the exponential function. In addition, two other higher order estimators were derived:

$$ {}^qD_i = (\sum_{ui} \tilde{p}_{ui}^q)^{(\frac{1}{1-q})} \quad \text{for } q \in \{2, 10\} \quad \text{and } \forall i \in \{i|S_i \geq 1\}, \tag{2} $$

where $q = 2$ is based on the reciprocal Simpson index, which gives less weight to rare species and more to common ones, while $q = 10$ primarily covers evenness by discarding most rare species and heavily weighting common ones (Hill, 1973; Pellens and Grandcolas, 2016).

To obtain relative abundances $\tilde{p}_{ui}$, systems of nonlinear equations were used to adjust observed species and reveal unobserved ones, assuming a geometric distribution for the latter (Chao et al., 2015). These equations included the abundance of each observed individual species c_{ui}, total ungulate abundance n_i (Chao et al., 2015) in each block, the

probabilities of an individual belonging to either the observed or unobserved species (Zhang and Huang, 2007; Chao and Shen, 2010; Chao et al., 2015) and, for the unobserved species, the bias-corrected species richness estimate ${}^0D_i$ (Marcon, 2015).

References

Burnham, K. P. and W. S. Overton (1979). Robust estimation of population size when capture probabilities vary among animals. *Ecology 60*(5), 927–936.

Chao, A., T. C. Hsieh, R. L. Chazdon, R. K. Colwell, and N. J. Gotelli (2015). Unveiling the species-rank abundance distribution by generalizing the Good-Turing sample coverage theory. *Ecology 96*(5), 1189–1201.

Chao, A. and T.-J. Shen (2010). *Program SPADE: Species Prediction and Diversity Estimation. Program and User's guide.* Hsin-Chu, Taiwan: CARE.

Hill, M. O. (1973). Diversity and evenness: a unifying notation and its consequences. *Ecology 54*(2), 427–432.

Marcon, E. (2015, Oct). Practical estimation of diversity from abundance data. Working paper or preprint. Available at: https://hal-agroparistech.archives-ouvertes.fr/hal-01212435v2/document.

Pellens, R. and P. Grandcolas (2016). Phylogenetic diversity measures and their decomposition: A framework based on Hill numbers. In *Biodiversity Conservation and Phylogenetic Systematics: Preserving our Evolutionary Heritage in an Extinction Crisis*, pp. 141–172. Cham, Switzerland: Springer Nature.

Shannon, C. E. (1948). A mathematical theory of communication. *The Bell System Technical Journal 27*(3), 379–423.

Zhang, Z. and H. Huang (2007). Turing's formula revisited*. *Journal of Quantitative Linguistics 14*(2-3), 222–241.

S3: Modeling ungulate diversity and biomass

S3.1: Boosting models

The raw species richness of ungulates was modeled using the negative binomial type I likelihood (Johnson et al., 2005), denoted as

$$f_{nbinom}(S; \mathbf{x}, s, \mathbf{t}, \mathbf{z}, \boldsymbol{\beta}, \boldsymbol{\gamma}), \qquad S = 0, 1, 2, 3, \ldots \tag{1}$$

where S is species richness, $\mathbf{x}$ is the vector of explanatory variables, s is space, $\mathbf{t}$ is the vector of dummy variables with 2 categories each, depending on the survey year 1999 or 2002, and $\mathbf{z}$ is the vector of dummy variables with 4 categories each, depending on the location in the Mara Reserve or on the pastoral lands in combination with the survey year. The symbol $\boldsymbol{\beta}$ denotes the parameter vector of the regression coefficients and models the log mean number of species, and $\boldsymbol{\gamma}$ is the parameter vector of the regression coefficients and models the log dispersion parameter for the negative binomial type I likelihood. The expected response in the number of species is given by

$$E(S; \mathbf{x}, s, \mathbf{t}, \mathbf{z}, \boldsymbol{\beta}) = e^{(f(\mathbf{x}, s, \mathbf{t}, \mathbf{z}, \boldsymbol{\beta}))}, \tag{2}$$

where e is the exponential function. The parameter vector of the regression coefficients $\boldsymbol{\gamma}$ models the logarithmic dispersion or scale parameter, which is treated as a redundant or nuisance parameter to derive the raw species richness predictions.

Bias-adjusted ungulate species richness, excluding zeros, was modeled using the truncated negative binomial type I likelihood (Johnson et al., 2005), denoted as

$$f_{trnbinom}({}^0D; \mathbf{x}, s, \mathbf{t}, \mathbf{z}, \boldsymbol{\zeta}, \boldsymbol{\varkappa}), \qquad {}^0D = 1, 2, 3, \ldots \tag{3}$$

where ${}^0D$ is the bias-adjusted species richness (Section S2), $\boldsymbol{\zeta}$ is the parameter vector of the regression coefficients for the log mean number of species, and $\boldsymbol{\varkappa}$ is the parameter vector of the regression coefficients for the log dispersion parameter. The expected response is given by

$$E({}^0D; \mathbf{x}, s, \mathbf{t}, \mathbf{z}, \boldsymbol{\zeta}) = e^{(f(\mathbf{x}, s, \mathbf{t}, \mathbf{z}, \boldsymbol{\zeta}))}. \tag{4}$$

The bias-adjusted ungulate diversity estimates, based on Shannon (order 1), Simpson (order 2), or species evenness (order 10) were continuous and equal to or greater than one. In contrast, the ungulate biomass estimates were approximately continuous and non-negative. Ungulate diversity estimates contained many ones, ranging from 19% to 23%, while biomass had a high proportion of zeros: 18% to 23% for all ungulates, 51% to 62% for migratory ungulates, and 23% to 26% for nonmigratory ungulates. To account for these data characteristics, we applied the zero-adjusted gamma likelihood.

For simplicity, we denote

$$f_{zaga}(y; \mathbf{x}, s, t, \boldsymbol{\vartheta}, \boldsymbol{\iota}, \boldsymbol{\kappa})$$

$$= \begin{cases} f_{zaga}({}^qD^*; \mathbf{x}, s, \mathbf{t}, \mathbf{z}, \boldsymbol{\lambda}_q, \boldsymbol{\xi}_q, \boldsymbol{\nu}_q) \quad \text{if } y = {}^qD^*, {}^qD^* \geq 0, \\ \qquad \text{for } {}^qD^* \in \{{}^1D^*, {}^2D^*, {}^{10}D^*\} \qquad (5a) \\ f_{zaga}(B^X; \mathbf{x}, s, \mathbf{t}, \mathbf{z}, \boldsymbol{\upsilon}_X, \boldsymbol{\phi}_X, \boldsymbol{\omega}_X) \quad \text{if } y = B^X,\ B^X \geq 0, \\ \qquad \text{for } B^X \in \{B^T, B^M, B^N\}, \qquad (5b) \end{cases}$$

where

$${}^qD^* = {}^qD - 1, \tag{6}$$

${}^qD$ represents ungulate diversity (Equation 2 in Section S2) based on orders 1 (Shannon), 2 (Simpson) and 10 (species evenness), B^T is total biomass, B^M is biomass for migratory ungulates, and B^N is biomass for non-migratory ungulates,

${}^qD$ represents ungulate diversity (Equation 2 in Section S2) based on orders 1 (Shannon), 2 (Simpson) and 10 (species evenness), B^T is the total biomass, B^M is the biomass for migratory ungulates, and B^N is the biomass for non-migratory ungulates,

$$\boldsymbol{\vartheta} = \begin{cases} \boldsymbol{\lambda}_q & \text{if } y = {}^qD^* \\ \boldsymbol{\upsilon}_X & \text{if } y = B^X \end{cases}, \quad \boldsymbol{\iota} = \begin{cases} \boldsymbol{\xi}_q & \text{if } y = {}^qD^* \\ \boldsymbol{\phi}_X & \text{if } y = B^X \end{cases},$$

$$\boldsymbol{\kappa} = \begin{cases} \boldsymbol{\nu}_q & \text{if } y = {}^qD^* \\ \boldsymbol{\omega}_X & \text{if } y = B^X \end{cases},$$

where $\boldsymbol{\lambda}_q$ is the parameter vector of the regression coefficients for the log mean of the

gamma likelihood component, $\boldsymbol{\xi}_q$ is the parameter vector of regression coefficients for the log dispersion or scale parameter of the gamma likelihood component and $\boldsymbol{\nu}_q$ is the parameter vector of regression coefficients for the zero likelihood component of the zero-adjusted gamma likelihood to model ungulate diversity. Conversely, $\boldsymbol{\upsilon}_X$ is the parameter vector of the regression coefficients for the log mean of the gamma likelihood component, $\boldsymbol{\phi}_X$ is the parameter vector of regression coefficients for the log dispersion or scale parameter of the gamma likelihood component and $\boldsymbol{\omega}_X$ is the parameter vector for the zero likelihood component of the zero-adjusted gamma likelihood to model ungulate biomass.

The zero-adjusted gamma likelihood combines the binary likelihood function for modeling zeros and the gamma likelihood function for modeling non-zeros, according to

$$f_{zaga}(y; \mathbf{x}, s, \mathbf{t}, \mathbf{z}, \boldsymbol{\vartheta}, \boldsymbol{\iota}, \boldsymbol{\kappa})$$

$$= \begin{cases} f_{zero}(0; \mathbf{x}, s, \mathbf{t}, \mathbf{z}, \boldsymbol{\kappa}) & \text{for } y = 0 \quad (7a) \\ (1 - f_{zero}(0; \mathbf{x}, s, \mathbf{t}, \mathbf{z}, \boldsymbol{\kappa})) \times f_{gamma}(y; \mathbf{x}, s, \mathbf{t}, \mathbf{z}, \boldsymbol{\vartheta}, \boldsymbol{\iota}) & \text{for } y > 0, \quad (7b) \end{cases}$$

where f_{zero} is the binary likelihood function and f_{gamma} is the gamma likelihood function (Rigby et al., 2019). The log likelihood of the zero-adjusted gamma model is the sum of the binary log likelihood and the gamma log likelihood (Rigby et al., 2019).

Binary likelihoods estimate the expected probabilities of zero ungulate diversity minus unity or zero ungulate biomass π_y via

$$E(\pi_y; \mathbf{x}, s, \mathbf{t}, \mathbf{z}, \boldsymbol{\kappa}) = logit^{-1}(f(\mathbf{x}, s, \mathbf{t}, \mathbf{z}, \boldsymbol{\kappa})) \tag{8}$$

using the inverse logit transformation (Hothorn et al., 2011). Gamma likelihoods provide non-zero estimates of ungulate diversity minus unity or ungulate biomass by

$$E(y; \mathbf{x}, s, \mathbf{t}, \mathbf{z}, \boldsymbol{\vartheta}) = e^{f(\mathbf{x}, s, \mathbf{t}, \mathbf{z}, \boldsymbol{\vartheta})}, \tag{9}$$

where e is the exponential function. The parameter vector of the regression coefficients $\boldsymbol{\kappa}$ models the logarithmic dispersion or scale parameter in the gamma likelihood, which is a redundant or nuisance parameter to derive the predictions.

Using the zero-adjusted gamma likelihoods (Eqs. 7a and 7b), the expected non-zero ungulate diversity minus unity or ungulate biomass relative to the explanatory variables $\mathbf{x}$, space s and time $\mathbf{t}$ are given by

$$E(y; \mathbf{x}, s, \mathbf{t}, \mathbf{z}, \boldsymbol{\vartheta}, \boldsymbol{\kappa}) = (1 - E(\pi_y; \mathbf{x}, s, \mathbf{t}, \mathbf{z}, \boldsymbol{\kappa})) \times E(y; \mathbf{x}, s, \mathbf{t}, \mathbf{z}, \boldsymbol{\vartheta}). \tag{10}$$

The expected responses for ungulate diversity are derived from

$$E({}^qD; \mathbf{x}, s, \mathbf{t}, \mathbf{z}, \boldsymbol{\lambda}_q, \boldsymbol{\nu}_q) = E({}^qD^*; \mathbf{x}, s, \mathbf{t}, \mathbf{z}, \boldsymbol{\lambda}_q, \boldsymbol{\nu}_q) + 1. \tag{11}$$

S3.2: Decomposing regression functions

To estimate how ungulate diversity and biomass change with environmental variables in different scenarios, we used variable coefficient models (Hastie and Tibshirani, 1990). The regression functions were additively decomposed by

$$
\begin{aligned}
f(\mathbf{x}, s, \mathbf{t}, \mathbf{z}, \boldsymbol{\psi}) = \quad & \underbrace{\sum_{k \in A \setminus C \cup \{s\}} f_{envt}(x_k, \mathbf{t}, \psi_k)}_{\text{time-varying environmental component}} \quad + \\
& \underbrace{\sum_{k \in A \setminus \{s\},\, m \in C} f_{envz}(x_{k_m}, \mathbf{z}, \psi_{k_m})}_{\text{time- and location-varying environmental components}} \quad + \quad \underbrace{f_{st}(s, \mathbf{t}, \psi_s)}_{\text{spatio-temporal component}}
\end{aligned}
\tag{12}
$$

where

$$
\boldsymbol{\psi} = \begin{cases}
\boldsymbol{\beta}, \boldsymbol{\gamma} & \text{for } y = \; S \\
\boldsymbol{\zeta}, \boldsymbol{\varkappa} & \text{for } y = \; {}^{0}D \\
\boldsymbol{\lambda}_q, \boldsymbol{\xi}_q, \boldsymbol{\nu}_q & \text{for } y = \; {}^{q}D^{*} \\
\boldsymbol{\upsilon}_X, \boldsymbol{\phi}_X, \boldsymbol{\omega}_X & \text{for } y = B^{X},
\end{cases}
$$

and f_{envt} represents environmental components that vary with census year, f_{envz} denotes environmental components that vary with both census year and land use, and f_{st} are the spatiotemporal local components characteristic of the study area (Hothorn et al., 2011). All initial models included the full set of 28 variables A (Appendix S3: Tables S1-S8), with regression parameters for distance to reserve boundary and space s depending on survey

year, denoted by dummy variables $\mathbf{t}$. All other environmental variables, except distance to reserve boundary and space, a subset C of A, varied as a function of both location in the Mara Reserve or on pastoral lands and survey year, denoted by dummy variables $\mathbf{z}$. By decomposing the regression functions, we accounted for spatiotemporal variability in diversity or biomass of savanna ungulates and captured the variable effects of numerous environmental predictors with census year or land use (Hothorn et al., 2011).

S3.3: Fitting boosting models

To save computational time in cross-validation needed to find the optimal number of boosting iterations, a non-cyclic machine learning algorithm was used to fit boosting models. The algorithm minimizes the negative log-likelihood (called the loss function) of the chosen statistical distribution (Hothorn et al., 2011), given by

$$\frac{1}{|I|}\sum_{i\in I}\rho(y_i,\boldsymbol{\eta}_{\theta_{loglik}}(\mathbf{x}_i,s_i,\mathbf{t}_i,\mathbf{z}_i)), \tag{13}$$

where

$$I=\begin{cases}\{1,2,\ldots,|S|\} & \text{for } y_i = \ S_i\\ \{i|{}^0D_i\geq 1\} & \text{for } y_i = \ {}^0D_i\\ \{i|{}^qD_i^*\geq 0\} & \text{for } y_i = \ {}^qD_i^*\\ \{1,2,\ldots,|B^X|\} & \text{for } y_i = \ B_i^X,\end{cases}$$

ρ is the loss function, and

$$\boldsymbol{\eta}_{\theta_{loglik}} =$$
$$\begin{cases} (\eta_{\mu_{nbinom}}, \eta_{\sigma_{nbinom}}) & \text{for } \mathbf{y} = \ \mathbf{S} \\ (\eta_{\mu_{trnbinom}}, \eta_{\sigma_{trnbinom}}) & \text{for } \mathbf{y} = \ {}^{0}\mathbf{D} \\ (\eta_{\mu_{zaga}}, \eta_{\sigma_{zaga}}, \eta_{\pi_{zaga}}) & \text{for } \mathbf{y} = \ {}^{q}\mathbf{D}^{*} \text{ v } \mathbf{B}^{X} \end{cases}$$

are the prediction functions for the distribution parameters of the applied statistical distributions (Mayr et al., 2012). The parameter μ denotes the location, σ the scale and π the skewness of the distributions derived from

$$\begin{aligned} \mu_{loglik} &= e^{(\eta_{\mu_{loglik}})}, \\ \sigma_{loglik} &= e^{(\eta_{\sigma_{loglik}})}, \\ \pi_{loglik} &= logit^{-1}(\eta_{\pi_{loglik}}), \end{aligned} \tag{14}$$

where $logit^{-1}$ is the inverse logit link function (Mayr et al., 2012). The term $\rho(y_i, \boldsymbol{\eta}_{\theta_{loglik}}(\mathbf{x}_i, s_i, \mathbf{t}_i, \mathbf{z}_i))$ is the contribution of the i'th observation to the negative log-likelihood of the specified distribution (Hothorn et al., 2011).

Instead of fitting regression functions to the true outcomes, the boosting algorithm fits one base learner at a time to the negative gradient vector of the loss function (Thomas et al., 2018). The negative gradient vectors for a given boosting iteration are partial derivatives of the negative log-likelihood for each distribution parameter based on the additive predictor estimate of the previous iteration (Thomas et al., 2018). Each base learner fit is evaluated

and improved by boosting in subsequent iterations (Thomas et al., 2018).

The non-cyclic boosting algorithm selects the best-fitting base learner for each distribution parameter in each iteration (Thomas et al., 2018). The best fit is determined by the smallest residual sum of squares with the gradient vector among the available base learners. It is given by

$$o^*_{loglik,p} = \underset{o=1,\ldots,O_{loglik,p}}{argmin} \sum_{i \in I} (g_{loglik,p,i} - \hat{h}_{loglik,p,o}(\mathbf{x}_i, s_i, \mathbf{t}_i, \mathbf{z}_i))^2, \tag{15}$$

where $o^*_{loglik,p}$ is the index of the selected best-fitting base learner for the p'th distribution parameter of the likelihood, and $g_{loglik,p,i}$ denotes the i'th element of the gradient vector (Thomas et al., 2018). The term $\hat{h}_{loglik,o,p}(\mathbf{x}_i, s_i, \mathbf{t}_i, \mathbf{z}_i)$ represents the i'th estimate of the o'th base learner for the distribution parameter (Thomas et al., 2018).

The non-cyclic algorithm updates the base learner for the distribution parameter that gives the greatest reduction in negative log-likelihood (outer loss) among preselected learners. This is expressed as

$$p^*_{loglik} = \underset{p=1,\ldots,P_{loglik}}{argmin} \Delta\rho_{loglik,p}, \tag{16}$$

where $\Delta\rho_{loglik,p}$ denotes the reduction in the negative log-likelihood when updating the p'th distribution parameter (Thomas et al., 2018). The reduction in the negative log-likelihood is derived from

$$\begin{aligned} \Delta\rho_{loglik,p} = \sum_{i \in I} \rho(y_i, \hat{\eta}_{\theta_{loglik,p}}(\mathbf{x}_i, s_i, \mathbf{t}_i, \mathbf{z}_i)^{[m-1]} + \\ sl * \hat{h}_{o^*_{loglik,p}}(\mathbf{x}_i, s_i, \mathbf{t}_i, \mathbf{z}_i)), \end{aligned} \tag{17}$$

where $\hat{\eta}_{\theta_{loglik,p}}(\mathbf{x}_i, s_i, \mathbf{t}_i, \mathbf{z}_i)^{[m-1]}$ is the additive predictor from the previous iteration, sl is the step length or learning rate, and $\hat{h}_{o^*_{loglik,p}}(\mathbf{x}_i, s_i, \mathbf{t}_i, \mathbf{z}_i)$ is the new estimate for the p'th distribution parameter based on the preselected base learner $o^*_{loglik,p}$ (Thomas et al., 2018). The non-cyclic algorithm saves computational time in cross-validation because it only needs to find the optimal number of iterations to update all distribution parameters (Thomas et al., 2018). In contrast, cross-validation with the cyclic algorithm must determine the optimal combination of iteration counts to update each distribution parameter (Thomas et al., 2018).

References

Hastie, T. J. and R. J. Tibshirani (1990). Modelling interactions. In *Generalized additive models*, pp. 264–280. London, New York, Tokyo, Melbourne, Madras: Chapman and Hall.

Hothorn, T., J. Müller, B. Schröder, T. Kneib, and R. Brandl (2011). Decomposing environmental, spatial, and spatiotemporal components of species distributions. *Ecological Monographs 81*(2), 329–347.

Johnson, N. L., A. W. Kemp, and S. Kotz (2005). Negative binomial distribution. In D. J. Balding, N. A. Cressie, N. I. Fisher, I. M. Johnstone, J. Kadane, G. Molenberghs, L. M. Ryan, D. W. Scott, A. F. Smith, and J. L. Teugels (Eds.), *Univariate Discrete Distributions* (3 ed.)., Chapter 5, pp. 231–232. Hoboken, New Jersey, USA: John Wiley & Sons.

Mayr, A., N. Fenske, B. Hofner, T. Kneib, and M. Schmid (2012). Generalized additive models for location, scale and shape for high-dimensional data - a flexible approach

based on boosting. *Journal of the Royal Statistical Society, Series C - Applied Statistics 61*(3), 403–427.

Rigby, R. A., M. D. Stasinopoulos, G. Z. Heller, and F. De Bastiani (2019). 20.1 Zero-adjusted gamma: ZAGA. In J. M. Chambers, T. Hothorn, D. Temple Lang, and H. Wickham (Eds.), *Distributions for modeling location, scale, and shape: Using GAMLSS in R*, Chapter Mixed distributions on [0, ∞), pp. 455–458. Boca Raton, London, New York: Chapman and Hall/CRC.

Thomas, J., A. Mayr, B. Bischl, M. Schmid, A. Smith, and B. Hofner (2018). Gradient boosting for distributional regression: faster tuning and improved variable selection via noncyclical updates. *Statistics and Computing 28*(3), 673–687.

S4: Extended results

S4.1: Correlations among predictors

Most of the 21 continuous predictors, 68% to 83%, were weakly correlated with each other (absolute Spearman's correlation coefficient less than 0.3; Appendix S1: Figures S4-S11). A minority of the 21 continuous predictors, 5% to 9%, had moderate correlations (absolute Spearman's correlation coefficient at least 0.5; Appendix S1: Figures S4-S11). Cattle tended to be associated with sheep and goats (Cramer's V: 0.31-0.46), but other associations between the categorical variables were weak (Figures S12-S15). Correlations between rainfall components were higher on the pastoral lands ($0.75 \geq r_s \leq 0.95$) than in the Mara Reserve ($0.32 \geq r_s \leq 0.81$; Appendix S1: Figures S4-S7). In the reserve, wet season rainfall increased towards the interior in 2002 ($r_s = -0.67$; Appendix S1: Figure S6), while distance to water decreased with wet season rainfall ($r_s = -0.5$) but increased with elevation ($r_s = -0.5$; Appendix S1: Figure S4). The preceeding month's rainfall also increased towards the interior of the reserve in both census years ($-0.67 \geq r_s \leq -0.64$; Appendix S1: Figures S4, S6). Seasonal rainfall components decreased with increasing elevation in the reserve in 1999 ($-0.57 \geq r_s \leq -0.56$; Appendix S1: Figure S4). Grass color increased with increasing rainfall in the preceeding month on both land uses in 1999 ($0.57 \geq r_s \leq 0.64$; Appendix S1: Figures S4, S5), and shrub color increased with increasing rainfall in the preceding month in the reserve in 1999 ($r_s = 0.53$; Appendix S1: Figure S4). Grass color also increased with seasonal rainfall on the pastoral lands in 1999 ($0.65 \geq r_s \leq 0.66$; Appendix S1: Figure S5). Tree cover increased with the preceeding month's rainfall on the pastoral lands in 2002 ($r_s = 0.56$; Appendix S1: Figure S7) and with increasing wet season rainfall

in the reserve in 1999 ($r_s = 0.52$; Appendix S1: Figure S4).

Grass height increased with grass cover on both land uses in 2002 ($0.54 \geq r_s \leq 0.57$; Appendix S1: Figures S6, S7). In the Mara Reserve, tree color was associated with tree height in both census years ($0.68 \geq r_s \leq 0.78$; Appendix S1: Figures S4, S6) and tree cover in 1999 ($r_s = 0.62$; Appendix S1: Figure S4). Shrubs were associated with trees ($0.56 \geq r_s \leq 0.61$), while shrub and tree color increased simultaneously ($0.51 \geq r_s \leq 0.69$) on the pastoral lands in both census years and in the Mara Reserve in 2002 (Appendix S1: Figures S4-S6). Shrub height increased with increasing shrub cover on both land uses in 1999 ($0.55 \geq r_s \leq 0.73$, Appendix S1: Figures S4,S5).

Distance to occupied bomas was related to distance to infrastructure ($r_s = 0.51$) or abandoned bomas ($r_s = 0.57$) and increased with wet season rainfall ($r_s = 0.61$) in the Mara Reserve in 2002 (Appendix S1: Figure S11). The distance to occupied bomas in the reserve increased with distance to the boundary in both census years ($-0.75 \geq r_s \leq -0.66$; Appendix S1: Figures S8, S10) and with distance to infrastructure in 1999 ($r_s = 0.58$; Appendix S1: Figures S8).

S4.2: Species contributions to the biomass and abundance of savanna ungulates

Wildebeest and zebra contributed most to the ungulate biomass on both land uses and in both census years (Appendix: Figures 5a-d). But while wildebeest was the most abundant species in the Mara Reserve in both years, they were only the second or third most abundant species on the pastoral lands (Appendix: Figures 5a-d, 4a-d). Zebra was the second largest contributor to the wild ungulate biomass in the Mara Reserve (Appendix:

Figures 5a, c). On the pastoral lands, zebra contributed as much as wildebeest to ungulate biomass in 1999 and was the largest contributor in 2002 (Appendix: Figures 5b, d). But, except in the Mara Reserve, where it was the second most abundant species in 2002, it was only the fourth most abundant ungulate species (Appendix: Figures 4a-d). Elephants were the third largest contributor to biomass in the reserve, while impala held this position on pastoral lands (Appendix: Figures 5a-d).

Thomson's gazelle and impala were among the most abundant ungulates (Appendix: Figures 4a-d) but except for impala on the pastoral lands, they contributed 5% or less to the ungulate biomass (Appendix: Figures 5a-d). Warthog (*Phacochoerus africanus*), waterbuck (*Kobus ellipsiprymnus defassa*), reedbuck (*Redunca redunca*), dik-dik (*Rhynchotragus kirkii*), hartebeest (*Alcelaphus buselaphus cokii*), bushbuck (*Tragelaphus scriptus*), black rhinoceros (*Diceros bicornis*) and duiker (*Sylvicarpa grima*) each contributed less than 1% to the ungulate biomass (Appendix: Figures 5). They were also rare (Appendix: Figures 4a-d). Rhinoceros, reedbuck, duiker and bushbuck were not observed on the pastoral lands in 1999 (Appendix: Figure 4b). Other ungulates included hippopotamus (*Hippopotamus amphibius*), giraffe, topi (*Damaliscus korrigum jimela*), Grant's gazelle (*Nanger granti*) and eland (*Taurotragus oryx*; Appendix: Figures 5a-d, 4a-d).
