# Supplementary material for: Comparative Ungulate Diversity and Biomass Change With Human Use and Drought: Implications for Community Stability and Protected Area Prioritization in African Savannas
Source: Ecol Evol. 2025 Aug 28;15(9):e71946. doi: 10.1002/ece3.71946 (PMC12391912; doi:10.1002/ece3.71946)
Supplement: Supplementary file 3 — Appendix S3: ece371946‐sup‐0003‐AppendixS3.pdf. [file ECE3-15-e71946-s001.pdf]

Appendix S3

Comparative ungulate diversity and biomass change with human use and drought: implications for community stability and protected area prioritization in African savannas

Ecology and Evolution

Gundula S. Bartzke, Joseph O. Ogutu, Hans-Peter Piepho, Claire Bedelian, Michael E. Rainy, Russel L. Kruska, Jeffrey S. Worden, Kamau Kimani, Michael J. McCartney, Leah Ng'ang'a, Jeniffer Kinoti, Evanson C. Njuguna, Cathleen J. Wilson, Richard Lamprey, N. Thompson Hobbs, Robin S. Reid

List of Tables

Table S1 2
Table S2 4
Table S3 6
Table S4 8
Table S5 10
Table S6 12
Table S7 14
Table S8 16

TABLE S1: The number of times each predictor variable was selected as a percentage of all the models applied to 51 complementary pairs of randomly selected subsets of data on the species richness of savanna ungulates in the Maasai Mara National Reserve and on the adjoining pastoral lands in Kenya for November 1999 and November 2002.

		**Percentage of selection in the total number of models**							
	**Year**	**1999**				**2002**			
	**Response**	μ_{NB}		σ_{NB}		μ_{NB}		σ_{NB}	
	**Landuse**	**Res.**	**Pas.**	**Res.**	**Pas.**	**Res.**	**Pas.**	**Res.**	**Pas.**
**Input variable**[a]	**Effect type**								
**Space**[b] (UTM coordinates)	smooth bivariate	**82**		32		**100**		13	
**Distance to the reserve boundary**[c]	smooth	0		0		**100**		**100**	
**Distance to water**[d]	smooth	**94**	**100**	2	0	**100**	**99**	0	0
**Distance to occupied boma**[d]	smooth	27	**100**	0	0	60	**100**	0	1
**Distance to abandoned boma**[d]	smooth	**100**	36	50	0	28	**100**	0	0
**Distance to infrastructure**[d]	smooth	14	41	0	0	53	**97**	0	0
**Wet season rainfall**[d]	smooth	**99**	**94**	17	0	34	13	0	0
Dry season rainfall[d]	smooth	4	0	7	0	24	0	0	0
**Preceding month's rainfall**[d]	smooth	20	25	0	0	7	**98**	0	0
**Slope**[d]	smooth	**99**	**91**	0	0	**100**	**99**	0	0
**Elevation**[d]	smooth	9	**100**	0	0	25	**99**	0	0

Continued on next page.

TABLE S1: Continued from preceding page.

		Percentage of selection in the total number of models							
	**Year**	**1999**				**2002**			
	**Response**	$\boldsymbol{\mu}_{NB}$		$\boldsymbol{\sigma}_{NB}$		$\boldsymbol{\mu}_{NB}$		$\boldsymbol{\sigma}_{NB}$	
	**Landuse**	**Res.**	**Pas.**	**Res.**	**Pas.**	**Res.**	**Pas.**	**Res.**	**Pas.**
**Input variable**[a]	**Effect type**								
**Grass cover**[d]	smooth	5	13	0	0	**98**	6	0	0
**Grass height**[d]	smooth	23	24	0	0	21	**99**	0	0
**Grass color**[d]	smooth	**100**	**80**	0	0	**99**	**100**	0	0
**Shrub cover**[d]	smooth	21	25	1	2	**100**	**71**	0	1
Shrub height[d]	smooth	14	15	0	0	41	44	0	0
Shrub color[d]	smooth	17	5	0	0	16	60	0	0
**Tree cover**[d]	smooth	**84**	**94**	0	0	64	38	0	0
**Tree height**[d]	smooth	16	12	0	0	28	**78**	0	0
**Tree color**[d]	smooth	**98**	**99**	0	0	**77**	21	0	0
**Presence of fire**[d]	linear	29	**72**	0	0	**91**	**83**	0	0
**Presence of sheep and goats**[d]	linear	0	65	0	0	**90**	**90**	0	0
Presence of cattle[d]	linear	20	4	0	0	19	13	0	0
Presence of carnivores[d]	linear	41	65	0	0	69	68	0	0
Presence of vehicles[d]	linear	40	14	0	0	55	27	0	0
Presence of litter[d]	linear	17	4	0	0	23	1	0	0

$\boldsymbol{\mu}_{NB}$, location parameter; and $\boldsymbol{\sigma}_{NB}$, scale parameter of the negative binomial distribution; Res., Reserve; Pas., Pastoral lands; [a]variables in bold were included in at least 70% of the models for the location parameter; $^{b}f_{st}$, $^{c}f_{envt}$ and $^{d}f_{envz}$ in Equation 12 in Supplementary material S2: Section S3.2.

TABLE S2: The number of times each predictor variable was selected as a percentage of all the models applied to 51 complementary pairs of randomly selected subsets of data on the biomass of savanna ungulates in the Maasai Mara National Reserve and on the adjoining pastoral lands in Kenya for November 1999 and November 2002.

		**Percentage of selection in the total number of models**											
	**Year**	**1999**						**2002**					
	**Response**	μ_{ZAGA}		σ_{ZAGA}		π_{ZAGA}		μ_{ZAGA}		σ_{ZAGA}		π_{ZAGA}	
	**Landuse**	**Res.**	**Pas.**	**Res.**	**Pas.**	**Res.**	**Pas.**	**Res.**	**Pas.**	**Res.**	**Pas.**	**Res.**	**Pas.**
**Input variable**[a]	**Effect type**												
**Space**[b] (UTM coordinates)	smooth bivariate	60		22		**100**		**74**		34		61	
**Distance to the reserve boundary**[c]	smooth	**100**		**100**		**95**		**96**		**100**		**100**	
**Distance to water**[d]	smooth	10	2	20	20	20	**78**	11	17	**72**	65	**81**	1
**Distance to occupied boma**[d]	smooth	44	23	8	**100**	0	25	10	**100**	**70**	**100**	0	**99**
**Distance to abandoned boma**[d]	smooth	34	34	61	44	**79**	0	6	15	24	68	0	3
**Distance to infrastructure**[d]	smooth	**97**	2	53	24	5	6	57	**99**	**75**	**80**	0	51
Wet season rainfall[d]	smooth	0	9	**81**	45	13	21	50	1	13	13	0	0
**Dry season rainfall**[d]	smooth	18	2	5	8	3	0	**90**	16	16	8	0	0
Preceding month's rainfall[d]	smooth	16	58	32	25	0	10	33	21	31	34	0	24
**Slope**[d]	smooth	4	0	32	**73**	**73**	37	20	3	**91**	58	10	0
**Elevation**[d]	smooth	67	**97**	47	**71**	0	9	0	**100**	28	46	0	3

Continued on next page.

TABLE S2: Continued from preceding page.

		Percentage of selection in the total number of models											
	**Year**	**1999**						**2002**					
	**Response**	μ_{ZAGA}		σ_{ZAGA}		π_{ZAGA}		μ_{ZAGA}		σ_{ZAGA}		π_{ZAGA}	
	**Landuse**	**Res.**	**Pas.**	**Res.**	**Pas.**	**Res.**	**Pas.**	**Res.**	**Pas.**	**Res.**	**Pas.**	**Res.**	**Pas.**
**Input variable**[a]	**Effect type**												
Grass cover[d]	smooth	14	34	**75**	31	0	2	36	12	**97**	15	25	0
Grass height[d]	smooth	37	0	55	37	14	0	65	0	69	17	2	21
**Grass color**[d]	smooth	**100**	67	52	12	69	4	**88**	38	**75**	46	0	0
**Shrub cover**[d]	smooth	**100**	36	**83**	44	10	2	30	31	13	18	26	12
Shrub height[d]	smooth	30	1	22	15	0	33	5	0	22	7	2	21
Shrub color[d]	smooth	2	0	18	22	4	4	20	13	33	53	0	0
**Tree cover**[d]	smooth	14	**85**	28	**85**	0	0	0	0	26	64	20	0
**Tree height**[d]	smooth	27	2	22	58	2	0	**100**	1	31	**98**	18	3
Tree color[d]	smooth	18	2	17	15	31	66	0	52	35	28	0	0
Presence of fire[d]	linear	0	0	30	12	0	0	1	19	49	**93**	5	0
Presence of sheep and goats[d]	linear	1	0	2	**74**	0	1	0	27	**93**	36	11	1
Presence of cattle[d]	linear	0	0	4	3	0	0	0	8	8	29	0	0
Presence of carnivores[d]	linear	1	15	22	36	0	0	1	43	28	63	0	0
Presence of vehicles[d]	linear	1	1	53	21	0	0	0	0	8	6	0	4
Presence of litter[d]	linear	11	1	47	14	12	0	0	2	67	56	0	0

μ_{ZAGA}, location parameter; σ_{ZAGA}, scale parameter and π_{ZAGA}, skewness parameter, of the zero adjusted gamma distribution; Res., Reserve; Pas., Pastoral lands; [a]variables in bold were selected in at least 70% of the models for the location parameter or the skewness parameter; [b]f_{st}, [c]f_{envt} and [d]f_{envz} in Equation 12 in Supplementary material S2: Section S3.2.

TABLE S3: The number of times each predictor variable was selected as a percentage of all the models applied to 51 complementary pairs of randomly selected subsets of data on the biomass of migratory savanna ungulates in the Maasai Mara National Reserve and on the adjoining pastoral lands in Kenya for November 1999 and November 2002.

		**Percentage of selection in the total number of models**											
	**Year**	**1999**						**2002**					
	**Response**	μ_{ZAGA}		σ_{ZAGA}		π_{ZAGA}		μ_{ZAGA}		σ_{ZAGA}		π_{ZAGA}	
	**Landuse**	**Res.**	**Pas.**	**Res.**	**Pas.**	**Res.**	**Pas.**	**Res.**	**Pas.**	**Res.**	**Pas.**	**Res.**	**Pas.**
**Input variable**[a]	**Effect type**												
**Space**[b] (UTM coordinates)	smooth bivariate	55		1		31		**91**		32		0	
**Distance to the reserve boundary**[c]	smooth	**100**		20		38		**91**		**100**		**99**	
**Distance to water**[d]	smooth	47	4	69	11	5	**82**	14	1	25	30	**73**	**87**
**Distance to occupied boma**[d]	smooth	62	3	22	24	0	**99**	68	**99**	**92**	**84**	3	**100**
Distance to abandoned boma[d]	smooth	6	28	10	11	25	8	7	8	11	47	2	46
**Distance to infrastructure**[d]	smooth	**100**	9	**98**	69	**95**	3	**94**	**98**	**75**	**71**	19	**94**
**Wet season rainfall**[d]	smooth	0	20	20	8	24	67	**93**	2	27	**71**	0	1
**Dry season rainfall**[d]	smooth	7	6	30	47	0	0	14	10	59	6	**86**	4
Preceding month's rainfall[d]	smooth	56	24	59	11	12	66	52	53	21	6	1	17
Slope[d]	smooth	4	15	8	3	7	0	15	34	6	41	16	36
**Elevation**[d]	smooth	14	13	64	3	12	**100**	3	**84**	6	41	17	56

Continued on next page.

TABLE S3: Continued from preceding page.

		Percentage of selection in the total number of models											
	Year	1999						2002					
	Response	μ_{ZAGA}		σ_{ZAGA}		π_{ZAGA}		μ_{ZAGA}		σ_{ZAGA}		π_{ZAGA}	
	Landuse	Res.	Pas.	Res.	Pas.	Res.	Pas.	Res.	Pas.	Res.	Pas.	Res.	Pas.
**Input variable**[a]	**Effect type**												
Grass cover[d]	smooth	19	30	28	22	0	4	38	8	53	23	48	2
**Grass height**[d]	smooth	6	1	25	38	**75**	0	20	0	**79**	4	1	57
**Grass color**[d]	smooth	**100**	**93**	64	11	**100**	41	61	1	35	30	**74**	42
**Shrub cover**[d]	smooth	**86**	3	62	16	6	26	22	6	8	48	**100**	**98**
Shrub height[d]	smooth	18	9	24	14	60	5	9	1	**80**	32	6	1
**Shrub color**[d]	smooth	1	5	25	5	0	0	1	**75**	41	36	6	0
Tree cover[d]	smooth	11	1	23	7	4	3	0	12	6	43	1	1
**Tree height**[d]	smooth	39	1	25	53	2	4	**99**	33	64	36	48	18
Tree color[d]	smooth	10	1	20	37	37	42	11	5	56	23	0	48
**Presence of fire**[d]	linear	0	0	0	37	0	7	8	1	49	17	38	**83**
Presence of sheep and goats[d]	linear	1	0	18	2	0	15	4	0	9	4	11	27
Presence of cattle[d]	linear	6	2	10	15	1	0	53	7	66	10	0	0
Presence of carnivores[d]	linear	1	0	10	23	0	19	2	22	24	**73**	2	46
Presence of vehicles[d]	linear	1	0	23	15	5	0	3	4	25	6	1	0
Presence of litter[d]	linear	20	3	16	25	0	1	8	0	44	3	1	0

μ_{ZAGA}, location parameter; σ_{ZAGA}, scale parameter and π_{ZAGA}, skewness parameter, of the zero adjusted gamma distribution; Res., Reserve; Pas., Pastoral lands; [a]variables in bold were selected in at least 70% of the models for the location parameter or the skewness parameter; [b]f_{st}, [c]f_{envt} and [d]f_{envz} in Equation 12 in Supplementary material S2: Section S3.2.

TABLE S4: The number of times each predictor variable was selected as a percentage of all the models applied to 51 complementary pairs of randomly selected subsets of data on the biomass of nonmigratory savanna ungulates in the Maasai Mara National Reserve and on the adjoining pastoral lands in Kenya for November 1999 and November 2002.

		**Percentage of selection in the total number of models**											
	**Year**	**1999**						**2002**					
	**Response**	μ_{ZAGA}		σ_{ZAGA}		π_{ZAGA}		μ_{ZAGA}		σ_{ZAGA}		π_{ZAGA}	
	**Landuse**	**Res.**	**Pas.**	**Res.**	**Pas.**	**Res.**	**Pas.**	**Res.**	**Pas.**	**Res.**	**Pas.**	**Res.**	**Pas.**
**Input variable**[a]	**Effect type**												
**Space**[b] (UTM coordinates)	smooth bivariate	15		**71**		**100**		0		**100**		**98**	
**Distance to the reserve boundary**[c]	smooth	27		**100**		46		18		**100**		**100**	
**Distance to water**[d]	smooth	**75**	0	33	47	10	62	25	12	**85**	**84**	47	1
**Distance to occupied boma**[d]	smooth	**75**	**97**	17	**72**	0	20	5	**100**	28	**99**	0	**99**
Distance to abandoned boma[d]	smooth	34	7	22	19	35	1	41	6	37	37	0	0
**Distance to infrastructure**[d]	smooth	24	4	29	24	4	1	31	**94**	40	49	1	46
Wet season rainfall[d]	smooth	10	0	**89**	29	14	16	3	43	5	19	0	0
Dry season rainfall[d]	smooth	9	0	14	4	2	1	0	35	21	2	1	1
Preceding month's rainfall[d]	smooth	6	13	22	0	0	16	21	7	64	**76**	0	12
**Slope**[d]	smooth	5	23	32	39	**77**	40	**73**	41	**70**	**66**	4	0
**Elevation**[d]	smooth	65	60	49	21	0	5	**100**	**97**	8	15	0	1

Continued on next page.

TABLE S4: Continued from preceding page.

		Percentage of selection in the total number of models											
	**Year**	**1999**						**2002**					
	**Response**	μ_{ZAGA}		σ_{ZAGA}		π_{ZAGA}		μ_{ZAGA}		σ_{ZAGA}		π_{ZAGA}	
	**Landuse**	**Res.**	**Pas.**	**Res.**	**Pas.**	**Res.**	**Pas.**	**Res.**	**Pas.**	**Res.**	**Pas.**	**Res.**	**Pas.**
**Input variable**[a]	**Effect type**												
**Grass cover**[d]	smooth	9	54	**79**	49	0	1	23	14	**98**	35	**70**	0
**Grass height**[d]	smooth	50	0	**83**	24	2	0	**87**	1	**91**	52	14	26
**Grass color**[d]	smooth	68	12	53	33	7	0	40	**98**	31	47	0	2
**Shrub cover**[d]	smooth	**98**	**84**	**84**	23	5	0	14	2	24	23	3	9
Shrub height[d]	smooth	35	44	64	44	0	9	7	3	15	6	10	2
**Shrub color**[d]	smooth	4	25	39	39	2	3	**92**	59	**80**	37	1	0
**Tree cover**[d]	smooth	20	**99**	48	**87**	2	0	49	53	62	25	5	0
**Tree height**[d]	smooth	14	9	33	24	3	0	5	**88**	48	26	**74**	0
Tree color[d]	smooth	23	8	23	41	52	53	24	24	35	28	1	0
Presence of fire[d]	linear	0	0	4	15	0	0	51	13	**68**	**92**	4	0
**Presence of sheep and goats**[d]	linear	0	8	0	60	0	0	0	**71**	**71**	18	0	0
Presence of cattle[d]	linear	0	1	12	33	0	0	26	0	9	40	0	
Presence of carnivores[d]	linear	3	10	28	11	2	0	0	53	**90**	25	0	0
Presence of vehicles[d]	linear	0	27	6	**68**	0	0	32	0	13	0	0	1
Presence of litter[d]	linear	5	1	11	4	9	0	2	1	36	45	0	0

μ_{ZAGA}, location parameter; σ_{ZAGA}, scale parameter and π_{ZAGA}, skewness parameter, of the zero adjusted gamma distribution; Res., Reserve; Pas., Pastoral lands; [a]variables in bold were selected in at least 70% of the models for the location parameter or the skewness parameter; [b]f_{st}, [c]f_{envt} and [d]f_{envz} in Equation 12 in Supplementary material S2: Section S3.2.

TABLE S5: The number of times each predictor variable was selected as a percentage of all the models applied to 50 complementary pairs of randomly selected subsets of data on the bias-adjusted species richness (^{0}D) of savanna ungulates in the Maasai Mara National Reserve and on the adjoining pastoral lands in Kenya for November 1999 and November 2002.

		**Percentage of selection in the total number of models**							
	**Year**	**1999**				**2002**			
	**Response**	μ_{TRNB}		σ_{TRNB}		μ_{TRNB}		σ_{TRNB}	
	**Landuse**	**Res.**	**Pas.**	**Res.**	**Pas.**	**Res.**	**Pas.**	**Res.**	**Pas.**
**Input variable**[a]	**Effect type**								
**Space**[b] (UTM coordinates)	smooth bivariate	29		0		**100**		0	
**Distance to the reserve boundary**[c]	smooth	3		0		**77**		3	
**Distance to water**[d]	smooth	54	**95**	0	0	**88**	**91**	0	0
**Distance to occupied boma**[d]	smooth	20	**100**	0	0	42	**100**	0	2
**Distance to abandoned boma**[d]	smooth	19	34	0	0	46	**100**	0	0
Distance to infrastructure[d]	smooth	58	52	0	0	65	69	0	0
**Wet season rainfall**[d]	smooth	**98**	59	0	0	44	6	0	0
Dry season rainfall[d]	smooth	0	6	0	0	15	1	0	0
**Preceding month's rainfall**[d]	smooth	63	40	0	0	24	**86**	0	0
**Slope**[d]	smooth	**91**	**91**	0	0	**100**	**80**	0	0
**Elevation**[d]	smooth	24	**100**	0	0	48	**100**	0	0

Continued on next page.

TABLE S5: Continued from preceding page.

		Percentage of selection in the total number of models							
	**Year**	**1999**				**2002**			
	**Response**	$\boldsymbol{\mu}_{TRNB}$		$\boldsymbol{\sigma}_{TRNB}$		$\boldsymbol{\mu}_{TRNB}$		$\boldsymbol{\sigma}_{TRNB}$	
	**Landuse**	**Res.**	**Pas.**	**Res.**	**Pas.**	**Res.**	**Pas.**	**Res.**	**Pas.**
**Input variable**[a]	**Effect type**								
**Grass cover**[d]	smooth	25	25	0	0	**98**	30	0	0
Grass height[d]	smooth	54	62	0	0	43	67	0	0
**Grass color**[d]	smooth	**99**	65	0	0	**100**	**100**	0	0
**Shrub cover**[d]	smooth	**70**	**72**	0	0	**99**	55	0	0
Shrub height[d]	smooth	37	11	0	0	39	25	0	0
**Shrub color**[d]	smooth	35	45	0	0	32	**75**	0	0
**Tree cover**[d]	smooth	**94**	**100**	0	0	48	62	0	0
**Tree height**[d]	smooth	**73**	45	0	0	18	**97**	0	0
**Tree color**[d]	smooth	**92**	**88**	0	0	**71**	45	0	0
**Presence of fire**[d]	linear	14	15	0	0	59	**79**	0	0
**Presence of sheep and goats**[d]	linear	9	62	0	0	69	**94**	0	0
Presence of cattle[d]	linear	23	34	0	0	57	57	0	0
Presence of carnivores[d]	linear	28	20	0	0	37	58	0	0
Presence of vehicles[d]	linear	18	28	0	0	39	13	0	0
**Presence of litter**[d]	linear	18	35	0	0	**72**	3	0	0

$\boldsymbol{\mu}_{TRNB}$, location parameter; and $\boldsymbol{\sigma}_{TRNB}$, scale parameter, of the truncated negative binomial distribution; Res., Reserve; Pas., Pastoral lands; [a]variables selected for the location parameter in at least 70% of the models are marked in bold; $^{b}f_{st}$, $^{c}f_{envt}$ and $^{d}f_{envz}$ in Equation 12 in Supplementary material S2: Section S3.2.

TABLE S6: The number of times each predictor variable was selected as a percentage of all the models applied to 50 complementary pairs of randomly selected subsets of data on the Shannon effective number of species (^{1}D) of savanna ungulates in the Maasai Mara National Reserve and on the adjoining pastoral lands in Kenya for November 1999 and November 2002.

		**Percentage of selection in the total number of models**											
	**Year**	**1999**						**2002**					
	**Response**	μ_{ZAGA}		σ_{ZAGA}		π_{ZAGA}		μ_{ZAGA}		σ_{ZAGA}		π_{ZAGA}	
	**Landuse**	**Res.**	**Pas.**	**Res.**	**Pas.**	**Res.**	**Pas.**	**Res.**	**Pas.**	**Res.**	**Pas.**	**Res.**	**Pas.**
**Input variable**[a]	**Effect type**												
**Space**[b] (UTM coordinates)	smooth bivariate	4		29		9		**94**		**100**		**100**	
**Distance to the reserve boundary**[c]	smooth	**98**		**100**		**100**		**86**		**100**		**100**	
**Distance to water**[d]	smooth	**92**	27	26	6	2	1	10	50	30	23	**80**	2
**Distance to occupied boma**[d]	smooth	6	28	12	27	3	**96**	16	**100**	19	28	0	**99**
**Distance to abandoned boma**[d]	smooth	48	6	69	40	8	0	38	**80**	16	40	1	0
**Distance to infrastructure**[d]	smooth	**94**	14	**88**	26	1	0	**96**	69	**94**	**80**	0	1
**Wet season rainfall**[d]	smooth	10	17	10	4	39	1	**86**	45	18	15	0	0
Dry season rainfall[d]	smooth	1	0	10	5	0	0	14	2	60	25	1	0
Preceding month's rainfall[d]	smooth	17	3	47	1	6	0	16	5	26	6	0	0
**Slope**[d]	smooth	17	51	13	67	**69**	1	49	**87**	51	41	0	0
**Elevation**[d]	smooth	7	**74**	43	0	0	**97**	**88**	**100**	**83**	53	0	24

Continued on next page.

TABLE S6: Continued from preceding page.

		Percentage of selection in the total number of models											
	**Year**	**1999**						**2002**					
	**Response**	μ_{ZAGA}		σ_{ZAGA}		π_{ZAGA}		μ_{ZAGA}		σ_{ZAGA}		π_{ZAGA}	
	**Landuse**	**Res.**	**Pas.**	**Res.**	**Pas.**	**Res.**	**Pas.**	**Res.**	**Pas.**	**Res.**	**Pas.**	**Res.**	**Pas.**
**Input variable**[a]	**Effect type**												
Grass cover[d]	smooth	56	17	48	8	0	0	47	51	**78**	40	23	0
Grass height[d]	smooth	52	19	31	23	15	1	9	21	**74**	10	0	0
**Grass color**[d]	smooth	**70**	8	22	15	16	0	**88**	**90**	50	**72**	10	2
**Shrub cover**[d]	smooth	**97**	31	**87**	48	4	0	**87**	27	**82**	36	27	0
**Shrub height**[d]	smooth	21	**91**	35	31	0	0	**86**	38	45	55	0	0
**Shrub color**[d]	smooth	35	8	11	31	1	0	4	**86**	22	37	6	0
**Tree cover**[d]	smooth	31	**95**	8	42	**81**	41	5	46	8	18	2	2
**Tree height**[d]	smooth	50	11	22	28	0	1	20	**93**	**71**	67	0	0
**Tree color**[d]	smooth	**70**	**79**	26	24	7	0	53	23	13	37	0	0
Presence of fire[d]	linear	0	34	9	1	1	0	4	14	3	18	10	0
**Presence of sheep and goats**[d]	linear	29	38	3	8	0	10	14	**84**	0	16	16	17
Presence of cattle[d]	linear	12	5	0	15	0	4	29	9	8	4	0	4
Presence of carnivores[d]	linear	12	2	15	26	1	0	21	57	37	21	0	0
Presence of vehicles[d]	linear	1	10	8	8	0	0	42	19	38	5	1	0
Presence of litter[d]	linear	12	36	20	18	0	0	6	9	29	14	1	0

$\boldsymbol{\mu}_{ZAGA}$, location parameter; $\boldsymbol{\sigma}_{ZAGA}$, scale parameter and $\boldsymbol{\pi}_{ZAGA}$, skewness parameter, of the zero adjusted gamma distribution; Res., Reserve; Pas., Pastoral lands; [a]variables in bold were selected in at least 70% of the models for the location parameter or the skewness parameter; [b]f_{st}, [c]f_{envt} and [d]f_{envz} in Equation 12 in Supplementary material S2: Section S3.2.

TABLE S7: The number of times each predictor variable was selected as a percentage of all the models applied to 50 complementary pairs of randomly selected subsets of data on the Simpson effective number of species (^{2}D) of savanna ungulates in the Maasai Mara National Reserve and on the adjoining pastoral lands in Kenya for November 1999 and November 2002.

		**Percentage of selection in the total number of models**											
	**Year**	**1999**						**2002**					
	**Response**	μ_{ZAGA}		σ_{ZAGA}		π_{ZAGA}		μ_{ZAGA}		σ_{ZAGA}		π_{ZAGA}	
	**Landuse**	**Res.**	**Pas.**	**Res.**	**Pas.**	**Res.**	**Pas.**	**Res.**	**Pas.**	**Res.**	**Pas.**	**Res.**	**Pas.**
**Input variable**[a]	**Effect type**												
**Space**[b] (UTM coordinates)	smooth bivariate	10		28		9		**92**		**98**		**100**	
**Distance to the reserve boundary**[c]	smooth	**98**		**100**		**100**		**83**		**77**		**100**	
**Distance to water**[d]	smooth	**96**	11	27	10	2	2	6	56	32	21	**84**	5
**Distance to occupied boma**[d]	smooth	3	11	14	25	3	**98**	19	**100**	30	19	0	**100**
Distance to abandoned boma[d]	smooth	57	8	**70**	41	9	0	41	61	18	57	1	0
**Distance to infrastructure**[d]	smooth	**90**	12	**96**	48	1	2	**92**	**74**	**97**	**75**	0	1
**Wet season rainfall**[d]	smooth	6	13	11	5	41	3	**82**	39	24	21	0	0
Dry season rainfall[d]	smooth	5	0	10	8	0	0	9	0	54	19	1	0
Preceding month's rainfall[d]	smooth	13	6	56	5	7	0	19	2	24	13	0	0
**Slope**[d]	smooth	5	39	17	66	**71**	3	26	66	50	48	0	0
**Elevation**[d]	smooth	8	**88**	40	4	0	**99**	**75**	**99**	**82**	**71**	0	33

Continued on next page.

TABLE S7: Continued from preceding page.

		Percentage of selection in the total number of models											
	**Year**	**1999**						**2002**					
	**Response**	$\boldsymbol{\mu}_{ZAGA}$		$\boldsymbol{\sigma}_{ZAGA}$		$\boldsymbol{\pi}_{ZAGA}$		$\boldsymbol{\mu}_{ZAGA}$		$\boldsymbol{\sigma}_{ZAGA}$		$\boldsymbol{\pi}_{ZAGA}$	
	**Landuse**	**Res.**	**Pas.**	**Res.**	**Pas.**	**Res.**	**Pas.**	**Res.**	**Pas.**	**Res.**	**Pas.**	**Res.**	**Pas.**
**Input variable**[a]	**Effect type**												
Grass cover[d]	smooth	48	18	53	15	0	0	36	39	**78**	43	31	0
Grass height[d]	smooth	47	17	43	25	22	4	8	13	**88**	27	0	1
**Grass color**[d]	smooth	39	5	20	23	18	0	**81**	62	61	**79**	13	2
**Shrub cover**[d]	smooth	**89**	38	**89**	64	5	1	**91**	22	**94**	37	30	0
**Shrub height**[d]	smooth	12	**86**	53	58	0	2	**71**	26	63	59	0	0
**Shrub color**[d]	smooth	30	8	16	45	1	0	9	**81**	26	32	8	0
**Tree cover**[d]	smooth	10	**84**	24	47	**83**	56	10	10	19	27	3	0
**Tree height**[d]	smooth	48	9	21	28	1	2	30	**81**	64	64	0	0
Tree color[d]	smooth	61	48	51	22	10	0	28	29	13	33	0	0
Presence of fire[d]	linear	0	23	14	4	1	0	2	10	5	29	12	0
Presence of sheep and goats[d]	linear	28	37	6	9	0	11	9	62	0	11	20	21
Presence of cattle[d]	linear	12	4	4	15	0	6	24	6	11	9	0	7
Presence of carnivores[d]	linear	12	5	28	21	4	0	17	52	36	15	0	0
Presence of vehicles[d]	linear	1	4	6	6	0	0	31	8	33	3	1	0
Presence of litter[d]	linear	13	27	9	27	0	1	11	8	29	22	2	0

$\boldsymbol{\mu}_{ZAGA}$, location parameter; $\boldsymbol{\sigma}_{ZAGA}$, scale parameter and $\boldsymbol{\pi}_{ZAGA}$, skewness parameter, of the zero adjusted gamma distribution; Res., Reserve; Pas., Pastoral lands; [a]variables in bold were selected in at least 70% of the models for the location parameter or the skewness parameter; [b]f_{st}, [c]f_{envt} and [d]f_{envz} in Equation 12 in Supplementary material S2: Section S3.2.

TABLE S8: The number of times each predictor variable was selected as a percentage of all the models applied to 50 complementary pairs of randomly selected subsets of data on the effective number of species in terms of evenness (^{10}D) of savanna ungulates in the Maasai Mara National Reserve and on the adjoining pastoral lands in Kenya for November 1999 and November 2002.

		**Percentage of selection in the total number of models**											
	**Year**	**1999**						**2002**					
	**Response**	μ_{ZAGA}		σ_{ZAGA}		π_{ZAGA}		μ_{ZAGA}		σ_{ZAGA}		π_{ZAGA}	
	**Landuse**	**Res.**	**Pas.**	**Res.**	**Pas.**	**Res.**	**Pas.**	**Res.**	**Pas.**	**Res.**	**Pas.**	**Res.**	**Pas.**
**Input variable**[a]	**Effect type**												
**Space**[b] (UTM coordinates)	smooth bivariate	16		19		9		**89**		**97**		**100**	
**Distance to the reserve boundary**[c]	smooth	**96**		**99**		**100**		**76**		33		**100**	
**Distance to water**[d]	smooth	**95**	7	33	16	2	3	6	64	25	24	**86**	9
**Distance to occupied boma**[d]	smooth	3	7	29	22	4	**99**	27	**98**	41	22	0	**100**
Distance to abandoned boma[d]	smooth	67	7	62	37	11	1	52	57	20	63	1	0
**Distance to infrastructure**[d]	smooth	**87**	10	**98**	49	1	4	**78**	**82**	**97**	**70**	1	1
**Wet season rainfall**[d]	smooth	13	12	11	5	42	4	**82**	32	18	20	0	0
Dry season rainfall[d]	smooth	5	1	8	8	0	0	7	1	48	16	1	0
Preceding month's rainfall[d]	smooth	10	3	64	9	9	0	18	2	16	15	0	4
**Slope**[d]	smooth	4	57	18	66	**75**	4	21	58	53	51	0	0
**Elevation**[d]	smooth	11	**90**	45	9	0	**100**	**71**	**91**	**81**	**78**	0	37

Continued on next page.

TABLE S8: Continued from preceding page.

		Percentage of selection in the total number of models											
	Year	1999						2002					
	Response	μ_{ZAGA}		σ_{ZAGA}		π_{ZAGA}		μ_{ZAGA}		σ_{ZAGA}		π_{ZAGA}	
	Landuse	Res.	Pas.	Res.	Pas.	Res.	Pas.	Res.	Pas.	Res.	Pas.	Res.	Pas.
**Input variable**[a]	**Effect type**												
Grass cover[d]	smooth	43	19	55	17	1	0	41	34	**78**	37	39	0
Grass height[d]	smooth	42	17	44	28	23	4	7	17	**89**	30	0	1
**Grass color**[d]	smooth	25	6	21	26	20	1	**93**	29	53	**85**	15	4
**Shrub cover**[d]	smooth	**78**	61	**92**	60	5	3	**85**	31	**93**	36	36	1
**Shrub height**[d]	smooth	7	**79**	56	65	0	4	62	22	67	59	0	0
**Shrub color**[d]	smooth	28	7	27	39	1	0	13	**78**	26	30	10	0
**Tree cover**[d]	smooth	6	**72**	35	52	**83**	59	9	43	28	26	4	0
**Tree height**[d]	smooth	36	12	25	23	1	2	36	**78**	67	60	0	0
Tree color[d]	smooth	53	19	57	34	11	0	11	41	21	29	0	0
Presence of fire[d]	linear	0	18	14	7	1	0	7	10	7	35	17	3
Presence of sheep and goats[d]	linear	32	36	8	11	0	1	5	40	1	8	24	24
Presence of cattle[d]	linear	14	3	3	15	0	8	13	3	22	10	0	7
Presence of carnivores[d]	linear	23	18	25	19	4	0	17	52	31	14	0	0
Presence of vehicles[d]	linear	0	1	6	7	0	0	28	8	31	3	1	0
Presence of litter[d]	linear	13	22	7	28	0	1	12	4	30	22	3	0

μ_{ZAGA}, location parameter; σ_{ZAGA}, scale parameter and π_{ZAGA}, skewness parameter, of the zero adjusted gamma distribution; Res., Reserve; Pas., Pastoral lands; [a]variables in bold were selected in at least 70% of the models for the location parameter or the skewness parameter; [b]f_{st}, [c]f_{envt} and [d]f_{envz} in Equation 12 in Supplementary material S2: Section S3.2.
