# Supplementary material for: Comparative Ungulate Diversity and Biomass Change With Human Use and Drought: Implications for Community Stability and Protected Area Prioritization in African Savannas
Source: Ecol Evol. 2025 Aug 28;15(9):e71946. doi: 10.1002/ece3.71946 (PMC12391912; doi:10.1002/ece3.71946)
Supplement: Supplementary file 4 — Appendix S4: ece371946‐sup‐0004‐AppendixS4.pdf. [file ECE3-15-e71946-s002.pdf]

# Appendix S4

**Comparative ungulate diversity and biomass change with human use and drought: implications for community stability and protected area prioritization in African savannas**

Ecology and Evolution

Gundula S. Bartzke, Joseph O. Ogutu, Hans-Peter Piepho, Claire Bedelian, Michael E. Rainy, Russel L. Kruska, Jeffrey S. Worden, Kamau Kimani, Michael J. McCartney, Leah Ng'ang'a, Jeniffer Kinoti, Evanson C. Njuguna, Cathleen J. Wilson, Richard Lamprey, N. Thompson Hobbs, Robin S. Reid

# Well done!

The Mara count 2002 was truly a team effort in every sense of the word. The count owes its existence and success to the 22 vehicle counting teams, 3 aircraft counting teams, 20 organisations and 84 individuals who counted in the rain and mud and contributed vehicles, lodging, airplanes, communications equipment, food, t-shirts, writing and filming expertise, GIS and statistical analysis help, counting practice help and venue, fuel, website help, engineering expertise, and moral support. We were all overwhelmed with the support and good will given so freely by so many. We list the people here who contributed and we give this report to you: this is your hard work and your accomplishment! We say a very big 'thank you' to all involved. It was an honour and pleasure to work with you.

# Mara Count 2002 supporters

## Overall count sponsors

Michael Rainy family, Campfire Conservation, Narok County Council, Mara Wildlife Trusts, Koyiaki Group Ranch, Olkinyei Group Ranch, and the International Livestock Research Institute (ILRI).

## Vehicle sponsors

Bike Treks Ltd, Mara Conservancy, Mrigesh Kshatriya, Wildlife Works, Abercrombie and Kent, Grant & Cameron Safaris, Campfire Conservation Ltd, African Conservation Centre, Sirata Sirua Ltd, Swara Plains Corp, Amara Conservation Ltd, Kobo Safaris, Friends of Conservation, Anne Kent Taylor Foundation/ A.K. Taylor International, International Livestock Research Institute, Vuorio/Mikkonen, World Wide Fund for Nature, Explore Mara Ltd., Jeff & Jessica Worden, Russell Kruska, Robin Reid, Cathy Wilson, Basecamp Masai Mara.

## Local Maasai counters were from

Mara Conservancy, Koyiaki Group Ranch, Olkinyei Group Ranch, Narok County Council Reserve, and Ol Chorro Oiroua Group Ranch.

## Special teams

Mechanics team: Josphat Sananka, Kuna.

## Roving technical and public awareness team

Peter Naurori, Russell Kruska and Susan MacMillan.

## Stationary technical team

Jawoo Koo, John Owuor and Leah N'gan'ga.

## Flying teams

Tristan Voorspuy and Jeffrey Worden (water resources); Richard Lamprey (forest resources); Kenya Wildlife Service (total counts of elephants, giraffe, eland and buffalo); Directorate of Resource Surveys and Remote Sensing (DRSRS; systematic reconnaissance count of wildlife, livestock and people, 5 x 5 km broad resolution).

## Filming team

Rob and Sarah O'Meara.

## Nairobi support team

Lucille Kirori, Mohammed Baya, Tom Ouna.

## GIS and statistical analysis and writing teams

Meshak Nyabenge, Leah N'gan'ga, John Owuor, Russell Kruska, Joseph Ogutu, Jeffrey Worden, Michael Rainy, Mrigesh Kshatriya and Robin Reid.

## International news coverage

Chris Tomlinson (Associated Press).

## Science sponsor

Carlos Seré, David Taylor, Bruce Scott, Bill Thorpe, Getachew Engida, Susan Dewey and Ralph von Kauffman of ILRI's senior management team.

## Count practice sponsor

Game Ranching Ltd.

## Accommodation contributors

Ker and Downey Safaris Ltd; Heritage Group (Mara Intrepids and Voyagers Lodges); Conservation Corporation (Kichwa Tembo Lodge); Serena Hotels; Mara River Lodge; Rekero; Basecamp; Kenya Wildlife Service; Mada Hotels (Fig Tree Lodge).

## Cash contributors

ILRI, Transworld Safaris Kenya Ltd, Friends of Conservation (Helen Gibbons), Bush Homes, Patti Kristjanson, Bateleur Safaris, Anthony Cheffings, World Wide Fund for Nature, Robin Hurt Safaris.

## Fuel contributor

Governor's Camp.

## T-shirt contributors

Susan Macmillan, Russell Kruska, and John Edwards.

## Website development

Web Fundi Ltd and Ian Gray.

## Engineering contributors

William Anyika, Amos Ndegwa and Francis Icharia.

## Communications sponsors

Michael & Judy Rainy from Sirata Sirua, Explore Mara Ltd., Ol Kanjau Tented Camp, Amboseli, Ololepo Ltd (Ololepo Landowners Wildlife Conservation Co. Ltd.).

## Radio installation

Phil Tilley of High Plains Engineering.

## Software and hardware sponsors

International Livestock Research Institute, Nairobi, Kenya, and reduced rates on mapping software purchases (ArcView, ArcInfo and ArcPad) from the Environmental Systems and Resources Institute (ESRI), Redlands, California.

## Organisers

Michael McCartney, Kamau Kimani, Michael Rainy, Judy Rainy, Jeffrey Worden and Robin Reid.

## Counting Teams

Team 1: Nigel Arensen, Eoin Harris, Alice Corrigan, Duncan Lanoi.

Team 2: Edward Nkoitoi, Patrick Siparo, Kamau Kimani.

Team 3: Burtie Hancock; Joshua Naiguran; Mrigesh, Patricia and Kristina Kshatriya.

Team 4: Evanson Kariuki, David Laboo, Rob Dodson.

Team 5: Jonathan Narasha, Peter Kamanga, William Ole Siara, Andrew Muchiru.

Team 6: Bruce McConnell, David Langat, Daniel Mpatany, Josphat Sananka.

Team 7: Jonathan Naurori, Moses Koriata, Meshak Nyabenge, Michael McCartney.

Team 8: Godfrey Masinde, Eliud Wanakuta, Josiah Musau, Johnson Ole Sititiek.

Team 9: Philip Cheres, Judy Rainy, Tenke Ntagusa , Heidi Bergemann.

Team 10: Kelvin Lenaronkoito, Michael Rainy, Phillip Tilley.

Team 11: Larali Lesorgol, Rob Fallon, Lori Bergemann.

Team 12: Elvira Omboke, Cyrus Ngatia, Oliver Lugalia, Fred Atieno.

Team 13: John Tira, Duncan Totana, John Kibriro, Sara Tourville, Bernadette Graham.

Team 14: Jackson Rakwa, Joseph Kimani, Peter Kamau, Tonya Troxler.

Team 15: Elias Jama, James Kaigil, Edwin, Nicholas Ole Kamuaro, Robin Reid.

Team 16: John Rakwa, Paul Lemein, Krista Mikkonen, Ville Vuorio.

Team 17: Joseph Ole Temut, Charles Matankory, Nina Bhola, Cathy Wilson.

Team 18: Pakuo Lesorogol, Sauna Lemiruni, Jacob Mayiani, Francis.

Team 19: William, Amos Tininah, Tonkei Taek, Johan Stenkula, Catherine, Jennifer Kinoti, Fumi Mizutani.

Team 20: Seyia, Jessica and Jeffrey Worden.

Team X: Andrew Muchiru, James ole Kaigil, Jackson ole Rakwa, Ville Vuorio

These acknowledgements were reproduced with minor corrections from the Mara Count report (available from `http://www.maasaimaracount.org/reports/Maracount.pdf`, accessed on 2 December 2022).
